# Does dosage matter? Effects of results-based financing layered on top of less comprehensive direct facility financing in Tanzania

**DOI:** 10.1093/heapol/czag058

**Published:** 2026-05-13

**Authors:** Eskindir Loha, Vincent Somville, Jo Borghi, Peter Binyaruka, Ottar Mæstad

**Affiliations:** Chr Michelsen Institute, PO Box 6033, Bergen N-5892, Norway; Department of Global Public Health and Primary Care, University of Bergen, Årstadveien 17, 5009 Bergen, Norway; Chr Michelsen Institute, PO Box 6033, Bergen N-5892, Norway; Department of Economics, Norwegian School of Economics, Helleveien 30, 5045 Bergen, Norway; Department of Global Health and Development, London School of Hygiene and Tropical Medicine, Keppel Street, London WC1E 7HT, United Kingdom; International Institute for Applied Systems Analysis, Scholssplatz 1, A-2361 Laxenburg, Austria; Chr Michelsen Institute, PO Box 6033, Bergen N-5892, Norway; Ifakara Health Institute, PO Box 78373, Dar es Salaam, Tanzania; Chr Michelsen Institute, PO Box 6033, Bergen N-5892, Norway

**Keywords:** performance-based financing, results-based financing, direct health facility financing, health service delivery, service quality, service coverage, maternal and child health

## Abstract

Performance-based financing at health facility level has improved service delivery in many low- and middle-income countries. However, the high costs of implementing such schemes have prompted interest in less complex forms of direct health facility financing. This paper measures the effects of layering a full-blown performance-based financing scheme (results-based financing, RBF) on top of a less comprehensive direct financing scheme in Tanzania. This enables us to assess whether implementing a less comprehensive scheme exhausted the potential for financing reforms to improve service delivery, or whether there are significant gains from adding more resources and incentives to the scheme. We estimated the effects of RBF using a difference-in-differences approach. Over 4 years, we tracked 150 health facilities and more than 3000 households, equally divided between eight districts that implemented both schemes and six districts that implemented only the less comprehensive scheme. Strong positive trends were observed for most outcomes in both groups of districts. At the same time, RBF had positive and statistically significant effects on 14 of 24 directly incentivized outcomes and on 22 of 47 other outcomes, including on service coverage (e.g. prenatal and vaccination services), service quality (e.g. content of care for antenatal and delivery services, drug availability, communication, and responsiveness), and patient satisfaction. A negative effect was estimated for one outcome only (use of family planning method). Statistically significant effects of RBF ranged from −4.3 to 16.2 percentage points (average: 8.7 pp). Analysis of intermediary outcomes revealed that RBF had a positive effect on health worker job satisfaction. We conclude that dosage matters: comprehensive direct financing schemes—with more resources and incentives—can significantly improve service delivery beyond what is achieved by less comprehensive ones.

Key messages

- Performance-based financing can lead to notable improvements in health service quality and coverage also when other forms of direct facility financing are implemented at the same time.
- Performance-based financing improved 36 of 71 outcome variables, including early presentation of pregnant women for antenatal care services, measles, and BCG vaccination coverage and the quality of maternal care.
- Performance-based financing improved health workers’ satisfaction with working conditions, which may have contributed to the positive effects on service quality and coverage.

## Introduction

Since the late 2000s, performance-based financing (PBF) has been implemented in the health sector across many low- and middle-income countries. PBF was regarded as a promising tool to improve service delivery in health systems where quality was substandard, and coverage of essential health services was insufficient (Eichler et al. 2009).

A defining feature of PBF is that health workers and/or health facilities receive financial incentives based on predefined performance indicators, such as the number of patients served and the quality of the services provided. In addition, PBF involves increased autonomy for health facilities to manage financial resources. Historically, health facilities in many low- and middle-income countries have relied on in-kind inputs, primarily delivered through local government authorities and have often faced delays in obtaining medical supplies and upgrading infrastructure. PBF mitigates these hurdles by directly depositing financial resources into health facilities’ bank accounts and granting them autonomy in spending those funds. PBF also involves strengthening of routines for financial management, clearer focus on priority setting, planning and reporting, greater community engagement in health facility management, and more feedback to health workers on their collective performance (Witter et al. 2021).

A growing body of research shows that PBF has improved quality of care in many settings. It has also increased the use of certain health services in several countries, although some negative effects have also been reported (Diaconu et al. 2021). However, the high cost of implementing PBF—largely due to need for extensive data verification (Antony et al. 2017)—has led to increased interest in simpler forms of direct financing. These alternative schemes share many features with PBF but lack its full set of incentive mechanisms, though some incentives may still be included.

This raises the question of to what extent such simpler forms for direct health facility financing (DHFF) can realize the potential of such reforms to improve service delivery, or whether more comprehensive schemes offer significant additional benefits.

This paper contributes to this discussion by reporting the effects of a PBF scheme implemented in selected districts in Tanzania from 2016 onwards—known as results-based financing (RBF). A unique feature of this setting is that, at the same time, the government of Tanzania implemented another, less comprehensive financing reform across all health facilities in the country—known as direct health facility financing (DHFF). RBF and DHFF shared several key features: both involved direct funding to health facilities; greater autonomy in budgeting and spending; a stronger emphasis on planning, reporting, and financial management; increased feedback to health workers; greater community involvement in decision-making; and both included financial incentives. The main difference was that RBF included a more comprehensive incentive package and higher resource transfers than DHFF. Thus, we can assess whether higher ‘dosages’ in these dimensions led to significant additional gains.

It is not clear what to expect. First, if the effects of PBF are primarily driven by factors, such as increased autonomy, a shift in focus towards outputs produced, and greater community engagement, then strengthening incentives and increasing resource transfers may yield only small additional gains. Second, the response to increased incentives and resources may not be linear: even small incentives and resource transfers may fully exhaust the potential of these factors to drive behavioural change, or conversely, effects might only emerge beyond certain thresholds. Finally, the small empirical literature that touches upon the issue is inconclusive: In Zambia and Nigeria, introducing incentives while doubling resource transfers did not produce meaningful additional gains (Friedman et al. 2016, Khanna et al. 2021). In Rwanda, introducing incentives without increasing resource transfers had positive effects on health services (Basinga et al. 2011, Sherry et al. 2017), while this did not happen in Cameroon (de Walque et al. 2021).

We find that RBF had significant effects beyond those of DHFF. While we observe large improvements in service utilization and quality in districts that only implemented DHFF, the improvements were notably higher in districts that implemented both RBF and DHFF. This suggests that dosage matters: the less comprehensive DHFF reform was not able to exhaust the potential for direct financing reforms to improve service delivery. We are unable to clearly determine whether resources or incentives mattered most, but we provide suggestive evidence that additional resources were the most important factor.

The paper contributes to the literature in three ways. First, to our knowledge, this is the first study to examine the effects of PBF in a setting where a less comprehensive direct financing scheme—with fewer incentives and lower resource transfers—is also being implemented. This allows us to shed light on an important policy question: whether simpler versions of direct health financing fully exhaust the potential of such reforms to improve service delivery, or if significant gains can be achieved by increasing the dosage.

Second, the paper contributes to a growing body of literature on the effects of PBF (Diaconu et al. 2021), providing estimates of the effects on a broad range of variables, including 71 final outcomes and 15 intermediary outcomes. The RBF scheme in Tanzania is broadly similar to schemes that have been studied in Rwanda (Basinga et al. 2011), Zambia (Friedman et al. 2016), Burundi (Bonfrer et al. 2014), Afghanistan (Engineer et al. 2016), Tajikistan (Ahmed et al. 2023), Burkina Faso (Steenland et al. 2017), Cameroon (de Walque et al. 2021), the Republic of Congo (Zeng et al. 2018), and Nigeria (Khanna et al. 2021). One aspect that distinguished the Tanzanian RBF programme from most others is that it included stronger measures to increase institutional deliveries, as community health workers (CHWs) were incentivized to escort pregnant women for delivery. We show that this strategy did not yield the expected results.

Third, this paper complements previous research on PBF in Tanzania, which examined a pilot scheme in a region near the financial capital, Dar es Salaam (Binyaruka et al. 2015, Borghi et al. 2021). It demonstrates that PBF can be implemented with significant effects in more disadvantaged areas as well.

### Context and background

Tanzania has a population of 61.7 million (Ministry of Health (MoH) [Tanzania Mainland] Ministry of Health (MoH) [Zanzibar] National Bureau of Statistics (NBS) Office of the Chief Government Statistician (OCGS) and ICF 2022) and has been classified as a lower-middle-income country since 2020. Forty-five per cent of the population live under the USD 2.15 extreme poverty line, and 23% are undernourished (World Bank 2024). Child mortality and infant mortality have been declining to 43 and 33 per 1000 live births (MoH et al. 2022).

The country has 31 administrative regions. Decentralized health services are offered at district hospitals, health centres, and dispensaries. CHWs also provide some community-based services but are not on the government payroll. While the government operates most health facilities, a significant number are run by faith-based organizations or private for-profit entities.

Tanzania piloted PBF in the health sector from 2011 to 2015 through the ‘Pay for Performance (P4P)’ programme in the Pwani region (Borghi et al. 2013). Health facilities were incentivized to achieve predefined targets for service utilization and quality of care. Seventy-five per cent of payments was provided as bonuses to health workers, while 25% was spent by health facilities. The evaluation of the programme found a positive effect on the utilization of two out of eight incentivized services after 13 months (Binyaruka et al. 2015), but the effects diminished over time (Borghi et al. 2021).

### Results-based financing and direct health facility financing

Building on the learning from the P4P pilot, the RBF programme was implemented sequentially in nine regions between 2015 and 2021 (from 2016 in the districts studied here). Poor health indicators and high poverty levels were key criteria for selecting regions to participate in the RBF programme (MoHSW 2015). Public health facilities that met a minimum quality standard were eligible for the programme. Facilities that did not meet the standard received lump-sum transfers that would enable them to qualify within a short period.

The programme provided health facilities a fee-for-service for 14 health services, adjusted by a quality score based on 18 groups of quality indicators. Base payments ranged from 0.25 USD for outpatient consultations to 12.5 USD for deliveries (Supplementary Tables S1 and S2). Payments were made quarterly. While 75% of the payments were spent by the health facilities with considerably autonomy, 25% was provided as staff bonuses based on staff attendance and level of responsibility. A comprehensive system was established for data verification. CHWs also received performance incentives for three indicators, including the number of household visits and the number of women escorted to health facilities for delivery (Supplementary Table S3). Incentives were also provided at the district and regional management levels, as well as for the Medical Stores Department based on four supply chain indicators.

The average RBF payment per facility per year in our sample was USD 10 668 in 2017/18 (Binyaruka et al. 2024). The share spent by the facilities (75%) represents a significant addition to their operational budgets. With an average of 5.3 medical staff per facility, the remaining amount translates into an average annual bonus per health worker of around USD 500, which is in the order of magnitude of a monthly salary.

The average RBF payment per facility per year in our sample was USD 10 668 in 2017/18 (Binyaruka et al. 2024). The share spent by the facility (75%) represents a significant addition to the operational budget. With an average of 5.3 medical staff per facility, the remaining amount translates into an average annual bonus per health worker of around USD 500, which is in the order of magnitude of a monthly salary.

RBF payments were made largely as planned until the end of 2018, when a delay of more than 1 year occurred. However, as most health workers believed that the payments were merely delayed, not stopped, the incentive system’s effect largely remained in place (Mæstad et al. 2021).

DHFF was implemented in all health facilities in the country from the fiscal year 2017/18 (MoHCDGEC 2017, Kapologwe et al. 2019). The disbursement of funds directly from the government to health facilities represented a shift away from providing in-kind inputs through district authorities. While half of the allocation was based on catchment population and distance from district headquarters, the remainder was allocated based on six outpatient and maternal health service indicators, the use of modern family planning methods, and the availability of 10 tracer medicines (MoHCDGEC 2020, WHO 2022).

Compared to RBF, DHFF incentivized fewer indicators, did not include financial incentives for health workers or CHWs, provided facilities with somewhat less autonomy over resources, and involved less intensive data verification (annual verification of 25% of health facilities, compared to quarterly verification of all facilities with RBF) (Mæstad et al. 2021). On average, the disbursed amounts were about half of those provided through RBF (Binyaruka et al. 2024). Hence, RBF added more resources, more incentives, and more autonomy on top of what was provided through DHFF.

### The results-based financing theory of change

**Figure 1:**
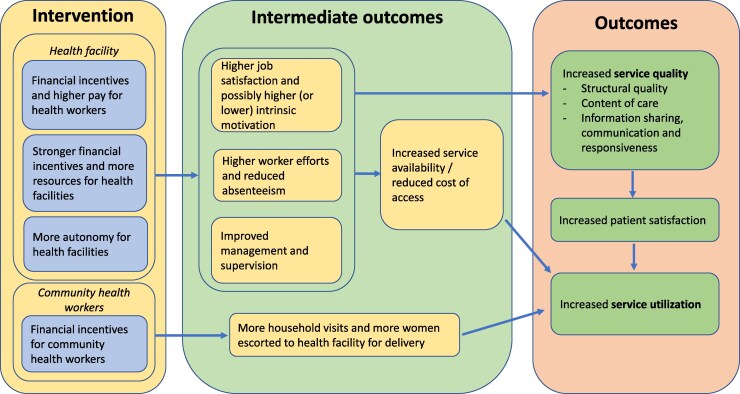
Theory of change.

Figure 1 outlines how RBF may have additional effects beyond what is achieved through DHFF alone.

Incentives for health workers may induce increased efforts to attract more patients by making services more accessible and/or by increasing service quality. Health workers had direct incentives to improve those quality indicators that are measured by the programme and indirect incentives to improve quality as perceived by clients, as improved client satisfaction may increase service utilization. As health worker bonuses were allocated partly based on attendance, health workers had direct incentives to be less absent from the workplace, which also may increase service availability and quality.

It may be rational for health workers to respond to incentives by putting less effort into nonincentivized services. Moreover, when incentives are provided for many services, as in this case, it may also be rational to reallocate efforts to services from some incentivized services to others where the gains are higher relative to the costs of effort (Holmstrøm et al. 1991). We do therefore not necessarily expect that incentives will increase quality and quantity for all incentivized services.

However, the RBF programme may affect performance through other mechanisms as well. First, since RBF payments increase the total resource envelope for health facilities, the programme enhances opportunities for increased service availability and quality. Second, more autonomy also enhances these opportunities by enabling more efficient resource utilization. Third, higher pay, more resources at the facility, and increased autonomy may boost health worker job satisfaction and motivation. This may counteract and possibly outweigh negative effects on nonincentivized or weakly incentivized services, as well as any potential negative effects of financial incentives on intrinsic motivation (Bènabou et al. 2006). Finally, incentives for health managers to supervise health facilities more regularly may also improve health worker performance.

## Methods

### Study design and data collection

We studied the effects of RBF using a controlled before and after study design. We compared changes over time in eight districts in Mwanza region, which implemented both RBF and DHFF, with those in six districts in Mara region, which implemented DHFF only. Comparison districts were selected by examining preintervention trends in key outcomes (e.g. institutional deliveries, four or more antenatal care (ANC) visits, three doses of the Diptheria-Pertussis-Tetanus vaccine (DPT3), and contraceptive use) and other related factors in districts across three regions that the government had identified as similarly disadvantaged as those in Mwanza. The pretrend analysis was conducted based on the Demographic and Health Survey (DHS) data between 1998 and 2010.

To further support the selection of districts of Mara region as a comparison, we compared each of the districts in Mara to those in Mwanza region based on outpatient visits, coverage of ANC, institutional deliveries, intermittent preventive treatment of malaria (IPT) during ANC, caesarean section rates, and measles immunization coverage for children under 1 year of age, using administrative data from 2014. We also considered the density of public health facilities per 1000 inhabitants and the mean quality score of health facilities, generated through the government’s ‘star rating’ system (Yahya et al. 2018), within each district. Each of these items was combined into an index generated through principal component analysis. We then compared the distribution of the index scores between districts that received the intervention and those that did not and found them to be broadly similar.

Health facilities were the primary sampling unit. We sampled 150 primary care facilities, 16 health centres, and 134 dispensaries, equally divided between the study arms. We first sampled health facilities in the Mwanza region, stratified across all districts and across facility quality scores (‘star ratings’). The aim was to achieve the same distribution of quality scores in our sample as observed in each of the districts. We used a matching approach to select facilities in the comparison region; for each health centre and dispensary in Mwanza, we identified the facility in Mara with the closest match.

Data on most outcome indicators were collected at the household level. From each facility’s catchment area, we sampled 20 (25) households at baseline (endline). Households were eligible if a woman in the household gave birth between 0 and 24 months before the survey. All neighbourhoods within a 5 km radius of the health facility were listed, and four of them were picked at random. A random walk method was employed to select an equal number of eligible households from each neighbourhood. In total, 2998 (3745) households participated in baseline (endline) surveys.

Baseline data were collected in February/March 2016, just before RBF implementation began, and endline data were collected in January/February 2020. At the time of the endline, implementation of RBF was about to start in the comparison districts. Collection of performance data began during the third quarter of 2019, but no RBF payments had yet been paid. To avoid contamination, our endline household survey did not include households with women that gave birth during the 6 months preceding the survey.

To explore the mechanisms through which RBF may work, we utilized three additional data sources: first, we conducted surveys with health workers and CHWs at baseline and endline. We aimed to interview two health workers and two CHWs at each facility. However, since not all facilities had more than one health worker present on the day of the data collection, the total sample consists of 273 (295) health workers and 207 (294) CHWs at baseline (endline).

Second, we conducted three rounds of phone interviews between February 2018 and November 2020 with health workers and CHWs who participated in the baseline survey, to monitor implementation fidelity and assess their understanding of the RBF scheme. The first two rounds included 134 health workers and 105 CHWs from the intervention region. The last round, which also included questions about DHFF, was conducted in both the intervention and comparison districts, comprising 294 health workers and 291 CHWs.

Finally, we did an in-depth, mixed-method process evaluation simultaneously with the endline survey in both intervention and comparison arms. These data were collected at one-third of the facilities in the main sample. Facilities were stratified into high and low baseline performance strata, based on their ‘star ratings’, and were then selected using proportionate random sampling. In this paper, we use this data set to report facility in-charges assessment of the relative importance of the various aspects of RBF for improved service delivery.

Overall, we measured 71 outcome indicators and 15 intermediary outcomes (Table 1).

**Table 1:** Summary of final and intermediary outcome variables and data sources.

	Number of variables		Data sources^a^
	Total	Directly incentivized	
Final outcomes			
Service utilization	10	3	HH surveys
Service quality			
Content of care	20	13	HH surveys
Structural quality	13	5	HF and HH surveys
Process quality	25	0	HH surveys
Patient satisfaction	3	2	HH surveys
Intermediary outcomes			
Job satisfaction/motivation	2	0	HW surveys
Health worker effort/absenteeism	3	1	HW and HF surveys
Management/supervision	3	0	HF and HW surveys
Service availability/copayments	6	0	HF and HH surveys
Community health worker effort	2	2	CHW surveys

^a^HH = household, HF = health facility, HW = health worker, CHW = community health worker.

Note that many indicators that are not directly incentivized are nevertheless indirectly incentivized. For instance, all 25 indicators of process quality were linked to ANC, delivery, or family planning services, which were directly incentivized. In the following, ‘incentivized services’ are those that are directly incentivized, while ‘nonincentivized services’ are those that are not directly incentivized.

### Measurement: health worker job satisfaction and motivation

Health workers’ satisfaction with working conditions was assessed using 11 items rated on a five-point Likert scale, while health worker motivation (or ‘personal drive’) was assessed using 16 items rated similarly. For both satisfaction and motivation, using the full set of items was rejected by confirmatory factor analysis. However, a subset of five items measuring job satisfaction and a subset of six items measuring health worker motivation performed well and were retained (Supplementary Tables S4 and S5).

The reliability of retained items in measuring the latent variables was assessed by calculating internal consistency using a polychoric correlation matrix (Gadermann et al. 2012, Borghi et al. 2018). The ordinal alpha was 0.71 for the five ‘satisfaction with work conditions’ items and 0.77 for the six ‘personal drive’ items.

To determine whether retained items measure the latent variables in the same way in both intervention and comparison groups, we assessed measurement invariance following the steps recommended by Putnick et al. (2016). The criteria for group comparison were satisfied for both measures (Supplementary Tables S6 and S7).

The unweighted sums of retained items were used in the difference-in-difference analysis.

### Data analysis

We measured the effects of RBF using a difference-in-differences (DID) approach. Effects on outcomes measured at the household level were estimated using a linear regression model with fixed effects:


$$Y_{ict}=\beta_{1}(\theta_{t})+\beta_{2}(X_{ict})+\beta_{3}(RBF_{c}\times \theta_{t})+\tau_{c}+\varepsilon_{ict},$$

where $Y_{ict}$ is the outcome for a woman in household *i* in facility catchment area *c*, at time *t*, $RBF_{c}\;$ is a dummy for the implementation of the RBF programme, $\theta_{t}$ is a time dummy, $X_{ict}\;$ is a vector of woman/household covariates, and $\tau_{c}$ is a health facility fixed effect. $\beta_{3}$ is the estimated effect of RBF.

The covariates $(X_{ict})\;$ include a household wealth index and individual characteristics of the interviewed woman, including her education, occupation, marital status, and number of living children. The wealth index was constructed from the following variables: ownership of a radio, mobile phone, table, sofa, toilet facility, and materials of floor and walls. Principal component factor analysis was conducted using oblique rotation. Two factors were retained, which explained 50% of the total variance. The Kaiser–Meyer–Olkin measure of sampling adequacy was 0.82, and the scale reliability coefficient was 0.71.

Standard errors were clustered at the facility level. Ideally, clustering should take place at a higher level, closer to the treatment level, but with only 14 districts, clustering at a higher level can be unreliable. As a robustness check, we also report district-level clustering using the wild cluster bootstrap approach.

To assess the validity of the parallel trend assumption, we examined trends for 64 outcome variables with available historical data over the 2-year period before the intervention, utilizing the variation in the timing of the most recent birth across households. The parallel trends hypothesis was rejected in only four cases, and in those cases, the deviations from parallel trends were extremely small (Supplementary Tables: Pre-trends).

Effects on facility-level outcomes were measured using a similar fixed effects DID approach, using robust standard errors and no covariates.

When measuring intermediary outcomes at the health worker level, we added the following covariates: years served in the facility and the health sector, growing up in the district, being in charge of the facility, medical/health training, age, and sex. For CHW intermediary outcomes variables, we used the following covariates: age, sex, native in the area, experience in years, any health training, trained recently, having weighing equipment, and mode of transport (foot, bicycle, motorcycle).

### Adjusting for multiple testing

We use Anderson’s code (Anderson 2008) to generate Benjamini, Krieger, and Yekutieli sharpened false discovery rate q-values (Benjamini et al. 2006). We adjusted for multiple testing within each category of outcome variables (service utilization, content of care, etc.).

## Results

### The added effects of results-based financing on aggregate service delivery indicators

Descriptive statistics of surveyed mothers, health workers, and CHWs are presented in Supplementary Tables S8–S10.

Table 2 summarizes the results by showing effects on aggregate service delivery indices. Two striking observations emerge. First, there is substantial progress on all aggregate indicators in comparison districts, with improvements ranging from 6.1 to 21.3 percentage points (pp). This suggests that substantial gains in service utilization and quality would have occurred even without RBF. Second, RBF has meaningful additional effects on all aggregate indicators, ranging from 3.3 to 9.4 pp, with greater impact on service quality than on service utilization. This suggests that dosage matters—additional resources and stronger incentives through the RBF programme significantly improved service delivery.

**Table 2:** Effects of RBF on aggregate service delivery indicator indices^a^ (%).

	Comparison		Intervention		DID estimation (95% CI)			
	Base	End	Base	End	Estimate	Lower	Upper	*P* value
Service utilization (10 items)	41.5	62.8*	40.9	65.0*	3.3*	0.7	5.9	0.015
Content of care (20 items)	57.0	70.0*	57.4	76.2*	5.7*	3.2	8.1	<0.001
Structural quality								
Enumerators’ observation (seven items^b^)	56.0	76.1*	54.1	83.7*	9.4*	2.5	16.3	0.007
Mothers’ observation (six items)	67.7	74.9*	70.0	83.2*	6.8*	3.7	9.9	<0.001
Process quality (25 items)	62.3	68.5*	59.3	71.7*	6.2*	3.0	9.4	<0.001
Client satisfaction (3 items)	72.0	82.0*	76.2	90.4*	5.1	−0.2	10.3	0.057

^a^Indices are the mean values of responses, in per cent. All responses are coded on a scale from 0 to 1.

^b^Some items are indices of underlying variables; 60 underlying variables are captured in total.

^*^Significant at 0.05 level (baseline–endline comparison and DID estimation).

### Disaggregating the added effects of results-based financing

Of the 71 outcome variables, 70 showed statistically significant improvement from baseline to endline in the intervention districts, while 61 showed improvement in the comparison districts (Tables 3–8).

**Table 3:** Effects on service utilization (%).

	Comparison		Intervention		DID estimation (95% CI)			
	Base	End	Base	End	Estimate	Lower	Upper	*P* value
Antenatal								
^a^ANC consultation before 12 weeks	21.1	30.8*	17.1	36.1*	10.6*	4.9	16.2	<0.001
^a^At least four ANC visits	60.9	78.7*	52.2	78.1*	7.7*	2.3	13.2	0.006
Delivery and postnatal								
^a^Institutional delivery	54.7	76.2*	68.4	89.4*	**−**0.3	−6.2	5.6	0.916
^a^Postnatal check-up within 3–7 days	65.9	73.4*	69.8	82.1*	4.6	−6.7	15.9	0.418
Child immunization and nutrition supplementation								
^a^Measles vaccination	68.0	82.9*	62.9	84.2*	8.7*	2.1	15.3	0.01
^a^Vitamin A supplementation	19.1	58.1*	20.8	59.2*	1.3	−6.2	8.7	0.733
BCG vaccination	92.2	98.3*	80.9	98.1*	11.8*	6.6	17.0	<0.001
DPTHibHepB3 vaccination	72.2	88.7*	72.7	91.8*	3.6	−2.0	9.2	0.211
Mebendazole	14.1	42.0*	18.0	41.7*	**−**0.0	−0.1	0.0	0.165
Family planning								
^a^Using any family planning method	18.4	32.8*	15.8	25.8*	**−**4.3*	−8.5	−0.1	0.045^b^

^a^Directly incentivized service.

^b^Sharpened q-value (*P* value adjusted for multiple testing) is above 0.05.

^*^Significant at 0.05 level (baseline–endline comparison and DID estimation).

**Table 4:** Effects on content of care (%).

	Comparison		Intervention		DID estimation (95% CI)			
	Base	End	Base	End	Estimate	Lower	Upper	*P* value
Antenatal care								
^a^Measured height	62.7	70.6*	69.6	83.6*	4.7	−1.6	11.1	0.143
^a^Took blood sample	84.8	93.1*	79.9	95.6*	7.0*	2.5	11.6	0.003
^a^Measured blood pressure	46.8	58.0*	55.1	74.3*	8.1*	0.7	15.6	0.032
^a^Listened to baby’s heart	97.5	98.0	95.5	99.0*	3.3*	1.3	5.4	0.002
^a^Gave or prescribed iron or folic acid	92.0	94.5*	87.1	96.5*	6.8*	3.1	10.4	<0.001
^a^Gave IPT, at least two doses	71.0	82.0*	66.0	83.8*	6.4*	0.4	12.5	0.037^b^
^a^Gave tetanus vaccination	70.7	74.5*	71.7	76.7*	**−**0.4	−5.5	4.6	0.865
Gave mebendazole	46.1	63.6 *	44.6	67.9*	5.1	−0.9	11.2	0.10
Measured weight	93.2	95.8*	93.5	97.7*	1.9	−2.0	5.8	0.337
Analysed urine	31.2	39.3*	34.1	51.6*	8.6*	1.6	15.7	0.017
Gave voucher for bed net	15.9	89.8*	21.5	95.6*	0.5	−4.2	5.1	0.85
Delivery care—the mother								
Checked blood pressure	19.0	36.4*	23.7	50.6*	9.5*	2.1	16.9	0.012
Took blood test	17.0	33.6*	18.5	45.2*	9.2*	1.8	16.6	0.015
Asked about abnormal bleeding	18.9	49.9*	22.6	58.9*	4.6	−2.0	11.2	0.174
Examined abdomen	63.7	72.7*	61.3	82.3*	13.4*	5.9	20.8	<0.001
Examined breasts	38.0	43.9*	35.4	55.7*	12.5*	3.2	21.9	0.009
Examined vagina	65.9	78.0*	60.8	83.7*	11.8*	3.6	20.1	0.005
Delivery care—the infant								
Weighed infant at birth	94.3	95.5	97.0	98.3*	**−**0.3	−2.7	2.2	0.828
Breastfeeding within an hour	40.0	60.5*	47.6	70.1*	3.0	−3.0	9.1	0.324
Vaccinated the infant	50.7	52.3	53.9	56.2	**−**0.9	−8.5	6.8	0.821

^a^Directly incentivized service.

^b^Sharpened q-value (*P* value adjusted for multiple testing) is above 0.05.

^*^Significant at 0.05 level (baseline–endline comparison and DID estimation).

**Table 5:** Effects on structural quality (%).

	Comparison		Intervention		DID estimation (95% CI)			
	Base	End	Base	End	Estimate	Lower	Upper	*P* value
Health facility characteristics (as observed by enumerators)								
^a^Drugs available (23 items)	57.6	65.2*	58.3	75.6*	9.7*	2.6	16.8	0.008
^a^Functional medical equipment (17 items)	59.1	80.6*	59.0	84.2*	3.7	−2.3	9.7	0.228
^a^Medical supplies (nine items)	53.6	72.3*	53.6	77.2*	4.9	−2.0	11.8	0.16
^a^Contraceptive supplies (eight items)	64.5	67.0	64.3	78.0*	11.2*	3.2	19.1	0.006
Electricity supply	46.7	73.3*	45.3	78.7*	6.7	−14.5	27.9	0.537
Improved water source (piped, well, pump)	37.3	81.3*	33.3	93.3*	16.0	−2.7	34.7	0.094
Functioning toilet (VIP or flush)	73.3	93.3*	65.3	98.7*	13.3	−2.8	29.5	0.105
Delivery care (as perceived by the mother)								
^a^The facility was not dirty	93.9	97.3*	89.2	97.4*	4.5*	1.1	8.0	0.01
^a^The delivery room was clean	95.4	97.6*	93.8	98.4*	2.7	−0.1	5.4	0.056
^a^The drugs the mother needed were available	52.9	68.0*	56.2	73.7*	3.2	−6.3	12.7	0.511
The hours the facility is open were adequate	94.2	93.9	88.8	96.2*	8.3*	4.1	12.4	<0.001
Any health service (as perceived by the mother)								
^a^Drugs available (last visit)	30.6	35.0*	34.2	54.4*	16.2*	9.7	22.8	<0.001
Facility found open (any visit during past 2 years)	84.0	85.9	82.1	86.8*	2.7	−2.9	8.3	0.345

^a^Directly incentivized indicator.

^*^Significant at 0.05 level (baseline–endline comparison and DID estimation).

**Table 6:** Effects on process quality—communication (%).

	Comparison		Intervention		DID estimation (95% CI)			
	Base	End	Base	End	Estimate	Lower	Upper	*P* value
Antenatal care—communication								
Discussed and advised on the place of delivery	81.6	93.9*	82.5	92.5*	**−**1.6	−6.1	2.9	0.486
Delivery care—communication								
Staff introduced themselves	19.7	31.5*	16.6	37.5*	9.5*	3.1	15.8	0.004
Asked if the mother wanted someone to support her during delivery	19.3	24.2*	20.5	33.5*	6.0*	0.3	11.7	0.04^a^
Explained what they were doing before conducting any procedure	26.0	34.2*	25.8	42.4*	7.9	−0.2	16.1	0.055
Advised what to do to make the mother more comfortable during pain	46.6	54.5*	43.4	64.5*	13.4*	4.2	22.6	0.004
Did a good job at explaining the progress of the delivery	77.9	89.3*	78.3	91.3*	1.6	−5.0	8.3	0.626
Discussed family planning	29.3	54.0*	33.3	55.2*	**−**3.7	−11.7	4.2	0.354
Talked about danger signs	17.8	36.2*	22.0	49.7*	8.0*	0.4	15.5	0.04^a^
Told the mother when to come back	52.7	58.0*	51.0	68.1*	11.3*	2.6	20.1	0.012
Gave advice about breastfeeding	35.7	53.6*	35.9	60.2*	3.5	−5.4	12.3	0.44
Discussed signs of newborn complications	20.2	32.3*	22.9	45.0*	9.7*	2.1	17.3	0.013
Family planning—communication								
Explained how FP methods work	76.9	85.7*	70.0	85.1*	4.4	−3.6	12.4	0.275
Explained the advantages and disadvantages of a particular method	68.8	73.7*	62.1	75.3*	7.1	−1.2	15.5	0.094
When the method of choice was not available, health worker told where she could receive it	83.8	97.2*	87.6	96.5*	**−**3.1	−8.5	2.4	0.27
Explained what to do in case of side effects	49.6	63.3*	45.7	70.5*	10.5*	1.2	19.9	0.027^a^

^a^Sharpened q-value (*P* value adjusted for multiple testing) is above 0.05.

^*^Significant at 0.05 level (baseline–endline comparison and DID estimation).

**Table 7:** Effects on process quality—responsiveness (%, unless stated otherwise).

	Comparison		Intervention		DID estimation (95% CI)			
	Base	End	Base	End	Estimate	Lower	Upper	*P* value
Delivery care—responsiveness								
Time spent with the health provider during the delivery was not too low	46.9	51.6*	46.4	53.4*	1.8	−6.6	10.1	0.672
Staff helped make the mother more comfortable during labour	60.9	71.9*	55.8	79.5*	12.5*	3.8	21.2	0.005
Staff came to assist the mother when she called for help	75.4	87.8*	74.8	86.6*	0.3	−6.1	6.8	0.919
Privacy was sufficiently respected	89.0	91.1	84.8	93.2*	7.2*	2.7	11.7	0.002
Was treated with respect and dignity	49.0	89.0*	49.1	81.5*	**−**6.7	−14.2	0.8	0.081
Staff’s kindness (mean, rated from 0 to 100)	76.3	84.4*	71.4	84.4*	5.0*	1.3	8.6	0.008
Any health service—responsiveness								
Staff took time to listen carefully	87.4	90.3	83.5	93.5*	7.7*	3.5	11.9	<0.001
No harsh words to the patients	76.0	79.2*	76.1	86.1*	6.9*	0.8	12.9	0.026
Treatment provided equally to rich and poor	79.5	87.8*	77.5	90.4*	5.8*	0.3	11.2	0.038
Waiting time less than 1 hour (last visit)	47.8	47.5	49.2	60.1*	12.2*	4.7	19.6	0.002

^*^Significant at 0.05 level (baseline–endline comparison and DID estimation).

**Table 8:** Effects on patient satisfaction (%).

	Comparison		Intervention		DID estimation (95% CI)			
	Base	End	Base	End	Estimate	Lower	Upper	*P* value
Delivery care								
^a^The overall quality of the service was satisfactory	88.0	92.2*	84.8	94.7*	5.4*	1.2	9.7	0.013
Mother would recommend the facility to friends	91.5	95.2*	90.6	96.3*	2.3	−1.4	5.9	0.22
Any health service								
^a^Satisfied with the overall quality of the service	66	71.3*	67.9	83.4*	10.9*	4.0	17.8	0.002

^a^Directly incentivized indicator.

^*^Significant at 0.05 level (baseline–endline comparison and DID estimation).

Amid significant general progress on almost all outcomes, we measured statistically positive effects of RBF on 36 of 71 outcome variables and a negative effect on one service utilization outcome (Table 2) [33 outcomes had significantly positive effects after adjusting for multiple testing (Supplementary Table S11)]. RBF had positive effects both on directly incentivized outcomes and on other outcomes. Among the 24 directly incentivized outcomes, RBF had statistically positive effects on 14 outcomes, a negative effect on one, and no effect on the remaining nine outcomes. The magnitude of statistically significant effects varied between −4.3 and 16.2 pp, with an average of 8.7 pp.

#### Service utilization

We assessed 10 service utilization variables, of which seven were directly incentivized (Table 3). RBF had positive effects on the utilization of antenatal and vaccination services. The proportion of mothers having ANC consultation before 12 weeks of gestation increased by 10.6 pp, and the proportion who had at least four ANC visits increased by 7.7 pp. These services were incentivized. When it comes to vaccinations, RBF increased measles vaccination (incentivized) by 8.7 pp and BCG vaccination (nonincentivized) by 11.8 pp.

There were no statistically significant effects on the following incentivized services: delivery at health facility, postnatal check-up within 3–7 days, and vitamin A supplementation for children. We estimated a negative effect on one incentivized service (current use of family planning method). The use of family planning increased significantly in both intervention and comparison districts, but more in the comparison districts. However, this effect was not significant when adjusting for multiple testing.

There was no decrease in service utilization of any nonincentivized services.

#### Content of care

We assessed 20 content-of-care items, of which seven were directly incentivized. Five incentivized and six nonincentivized outcomes improved due to RBF (Table 4). ‘Antenatal care’: most of the directly incentivized variables related to ANC improved—taking blood samples increased by 7.0 pp, measuring blood pressure by 8.1 pp, listening to the baby’s heart by 3.3 pp, receiving iron or folic acid prescription by 6.8 pp, and receiving at least two doses of IPT by 6.4 pp. Interestingly, the largest improvement in the content of ANC is seen for a nonincentivized service, urine analysis, which increased by 8.6 pp. ‘Delivery care’: we observe sizable positive effects on the content of delivery services, despite no direct incentives. The proportion who had blood pressure checked and blood tests taken increased by 9.5 pp and 9.2 pp. There was also a large increase in physical examination of the mother’s abdomen (13.4 pp), breasts (12.5 pp), and vagina (11.8 pp).

#### Structural quality

We assessed 13 indicators of structural quality, of which eight were directly incentivized. RBF resulted in statistically significant improvements in five indicators, four of which were directly incentivized (Table 5). Large effects are observed on drug availability; we monitored 23 drugs and measured an average increase in their availability of 9.7 pp. Women reported a 16.2 pp increase in drug availability at their last visit (any health service), but not any increase in the availability of the specific drugs they needed during their last delivery. Contraceptives are also more available on average (11.2 pp across eight items), while we do not observe any effects on the average availability of functional medical equipment (17 items) or medical supplies (nine items).

RBF also led to an increase in clients reporting that the facility was not dirty (4.5 pp) and that opening hours were adequate when they came for delivery (8.3 pp). There was, however, no effect on the general opening hours or on the availability of electricity, improved water, or functioning toilets.

#### Process quality—communication and responsiveness

None of the 25 indicators used to measure process quality were directly incentivized. RBF had a statistically positive effect on 14 items (Tables 6 and 7).

We observe particularly large effects for delivery services. Health workers introduce themselves to a larger proportion of the women who came to deliver (9.5 pp), discussed signs of newborn complications (9.7 pp), talked about danger signs (8.0 pp), advised how to become more comfortable during pain (13.4 pp), and explained what they were doing (7.9 pp). A larger proportion of the mothers also reported that their privacy was respected (7.2 pp), and the reported degree of staff kindness increased by an average of 5.0 points (rated on a scale from 0 = harsh/unkind to 100 = very kind).

Some improvement was also observed during family planning sessions, where a higher proportion heard the health worker explain what to do in the case of side effects (+10.5 pp).

Improved communication and responsiveness for particular services may result in less such inputs in other services. It is therefore interesting to observe improvements also when we ask about any health service that respondents have used; RBF resulted in a larger proportion experiencing that staff took time to listen carefully (+7.7 pp), did not utter harsh words (+6.9 pp), and treated the rich and the poor equally (+5.8 pp).

RBF also reduced waiting times; the proportion that had to wait for more than 1 hour was reduced by 12.2 pp.

#### Satisfaction with health services

RBF improved client satisfaction with the overall quality of health services by 10.9 pp (Table 8). Improvements in indicators measuring overall satisfaction with delivery services were not statistically significant, though. Note that these satisfaction indicators were high also at baseline, around 90%, limiting the scope for further improvement.

### Mechanisms: what may have contributed to the effects of results-based financing?

This section sheds further light on the causal mechanisms outlined in the theory of change. We start by discussing implementation fidelity and then present measured effects on intermediary outcome variables, supplemented by findings from the process evaluation. The data suggest that RBF improved health worker job satisfaction, mostly due to improved physical working conditions. CHWs increased their efforts by escorting many more women for delivery, but apparently only those who would have delivered at the facility anyway. A number of other intermediary outcomes improved both in intervention and comparison districts, but we could not document any differential effect of RBF. Facility in-charges point to increased resource availability as the main reason for the effects of RBF.

#### Implementation fidelity and knowledge of the results-based financing scheme

The RBF programme was largely implemented as planned. Some facilities did not participate from the start because they did not fulfil the minimum quality requirements, but they were quickly brought up to the required level. The implementation for CHWs was a bit slower; by 2018, 93% of health workers and 74% of CHWs reported that they had received a bonus (‘phone survey, round 2’). The main implementation challenge was a delay in payments that occurred in late 2018 when it took more than 1 year to release the funds. However, more than 90% of health workers claimed that service delivery continued like normal despite this delay and that health worker motivation was not reduced, while 78% claimed that drug supply was maintained (‘phone survey, round 3’). This suggests that while much went on as normal, there were also some reductions in structural quality in some facilities. CHWs more clearly expressed that delayed payments reduced their motivation; 33% stated that it reduced the number of household visits (‘phone survey, round 3’).

Health workers were not consciously aware of all the services that were incentivized; they were unable to list more than around half of the 14 incentivized services (‘phone survey, round 3’). With such limited awareness, we would not expect targeted efforts to increase utilization of all the incentivized services. CHWs, on the other hand, who had only three incentivized activities, were able to almost perfectly recall them all (‘phone survey, round 3’).

#### Health worker job satisfaction and motivation

RBF improved health worker job satisfaction. The estimated effect represents a 13.2% increase from baseline (Table 9). The effect is driven mainly by increased satisfaction with the physical condition of the facility. The score on this item increased by 42.6% in the intervention districts and 17.8% in the comparison districts (Supplementary Table S4). RBF did not affect our measure of motivation, or ‘personal drive’. There was a slight improvement in both intervention and comparison districts but no differential effect.

**Table 9:** Effects on health worker job satisfaction and motivation (score on five-point Likert scale).

	Comparison		Intervention		DID estimation (95% CI)			
	Base	End	Base	End	Estimate	Lower	Upper	*P* value
Satisfaction with working conditions (five items)^a^	2.65 (0.81)	3.24* (0.88)	2.80 (0.87)	3.73* (0.76)	0.37*	0.10	0.64	0.008
Personal drive (six items)^a^	3.32 (0.42)	3.45* (0.39)	3.27 (0.39)	3.44* (0.42)	0.05	−0.09	0.18	0.51

^a^Based on unweighted means of the items. See Supplementary Tables S4 and S5.

^*^Significant at 0.05 level (baseline–endline comparison and DID estimation).

#### Health worker efforts/absenteeism

**Figure 2:**
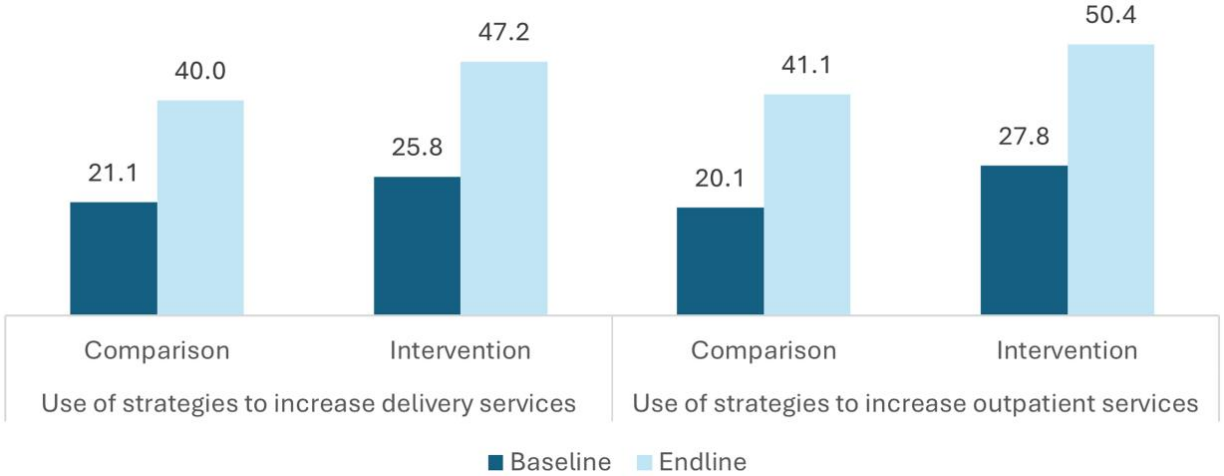
Reported use of strategies to increase service utilization during the past 12 months (%). Unweighted averages of the use of 10 strategies to increase delivery services and eight strategies to increase outpatient services.

As shown in Section 3, RBF caused health workers to increase their efforts in providing quality services, to the extent that this was duly noticed by the clients. However, it is less clear that RBF stimulated health workers to make more direct efforts to attract more patients to the facility, e.g. by urging CHWs to send more patients, urging people directly to come, improving transport services for pregnant women, or distributing free delivery kits. Figure 2 summarizes changes in the stated use of 18 specific strategies to increase the utilization of delivery and outpatient services. Health workers report a significant increase in the use of such strategies from baseline to endline, but there is no significant difference between intervention and comparison districts for 17 of the 18 strategies (for details, see Supplementary Table S12).

The formula for allocating RBF bonuses to health workers provided them with incentives to be less absent from the facility. However, our data do not provide support for a reduction in absenteeism. We measured absenteeism on the first day of data collection at health facilities and found no change between baseline and endline neither in intervention nor comparison districts (Table 10).

**Table 10:** Effects on absenteeism (%).

	Comparison		Intervention		DID estimation (95% CI)			
	Base	End	Base	End	Estimate	Lower	Upper	*P* value
Staff present at health facility	79.6	80.6	81.4	77.2	**−**5.3	−13.2	2.6	0.187

Nor did we observe any change in the staff composition as a result of RBF (Supplementary Table S13).

#### Management and supervision

There was a significant increase in the frequency of meetings of health facility governing committees (HFGCs) in both arms, but no change could be attributed to RBF (Table 11). Likewise, more than 90% of facility in-charges in both regions reported that the influence of HFGCs on decision-making had increased during the past 4 years.

**Table 11:** Effects on management and supervision (%, unless stated otherwise).

	Comparison		Intervention		DID estimation (95% CI)			
	Base	End	Base	End	Estimate	Lower	Upper	*P* value
Management								
Number of HFGC meetings in the past 12 months [mean (SD)]	3.8 (2.2)	5.0* (4.6)	3.4 (1.6)	5.7* (3.8)	1.1	−0.4	2.6	0.162
Supervision								
Met with supervisor during the past 90 days	56.6	91.3*	68.6	95.2*	**−**7.9	−20.6	4.9	0.225
Received feedback on the quality of work from external supervisor during the last meeting	87.0	98.0*	81.7	95.1*	**−**2.8	−13.2	7.6	0.595

HFGC = health facility governing committee.

^*^Significant at 0.05 level (baseline–endline comparison and DID estimation).

A similar pattern is observed for supervision: there are significant increases in the share of health workers who had been supervised during the past 90 days and had received feedback during supervision. But since the change happened in both intervention and comparison districts, it cannot be attributed to RBF.

#### Health service availability/copayments

There is no evidence of any increase in the formal opening hours of health facilities, neither for outpatient nor delivery services (Table 12). This contrasts with the experience of women who came for delivery, but the data are not necessarily inconsistent as there is considerable informality around opening hours for such services. There is, however, a significant increase in the number of facilities that provide outreach services, but this change cannot be attributed to RBF.

**Table 12:** Effects on service availability and copayments (%, unless stated otherwise).

	Comparison		Intervention		DID estimation (95% CI)			
	Base	End	Base	End	Estimate	Lower	Upper	*P* value
Health service availability								
Days a week open for outpatient services [mean (SD)]	5.3 (0.7)	5.4 (0.8)	5.4 (1.0)	5.6 (0.9)	0.1	−0.3	0.5	0.534
Facility offers 24-hour delivery services	86.5	85.3	89.3	88.0	**−**0.2	−15.4	15.1	0.981
Facility conducts outreach services	69.3	94.7*	78.7	98.7*	**−**5.3	−20.5	9.9	0.492
Copayments: delivery care								
Did not pay for delivery care services	73.1	87.1*	68.2	95.6*	11.8*	5.4	18.1	<0.001
Did not purchase supplies to bring for the birth	61.9	46.5*	42.7	32.4*	5.2	−1.7	12.1	0.135

^*^Significant at 0.05 level (baseline–endline comparison and DID estimation).

There is mixed evidence about the effects of RBF on copayments. According to the mothers, RBF increased the proportion that did not have to pay for delivery services by 11.8 pp (Table 12). More health workers also report that charges have been reduced for this service, but in the health worker data, this change is observed in both intervention and comparison districts and can therefore not be attributed to RBF (Supplementary Table S12). Note also that mothers in both intervention and comparison districts report that while fewer have to pay for delivery services, more women have to bring supplies for the birth (Table 12).

#### Efforts by community health workers

Self-reported data from CHWs suggest that RBF had a large positive effect on the number of women that they escorted for delivery (Table 13). In the intervention districts, the number of women escorted during the past 3 months increased from 2.5 to 11.4, a much larger increase than in the comparison districts. In phone interviews with health workers, more than 90% reported that CHWs escorted more women for delivery than before. But since RBF did not increase the number of women who delivered at facilities, the CHWs apparently escorted women who would have delivered at the facility anyway. RBF had no effect on the average number of household visits conducted by the CHWs.

**Table 13:** Effects on community health worker performance.

	Comparison		Intervention		DID estimation (95% CI)			
	Base	End	Base	End	Estimate	Lower	Upper	*P* value
Number of women escorted in the last 3 months	3.3 (7.7)	7.3* (7.5)	2.5 (4.9)	11.4* (11.1)	4.3*	1.3	7.3	0.005
Number of households visited last week	13.3 (26.7)	11.4 (15.5)	13.0 (28.1)	11.2 (11.0)	0.8	−7.6	9.3	0.849

^*^Significant at 0.05 level (baseline–endline comparison and DID estimation).

#### The role of financial resources and incentives

Data from the mixed-method process evaluation suggest that increased funds available to health facilities and stronger financial incentives were the aspects of RBF that contributed most to its effects (Table 14) [facility in-charges from the intervention districts who participated in the mixed-method process evaluation at endline were asked to indicate the relative importance of different aspects of RBF by allocating a given number of coins across each of these aspects (Mæstad et al. 2021)].

**Table 14:** Facility in-charges’ assessment of the relative importance of key elements of RBF for its effects on service delivery.

RBF element	Relative importance
More funds for the facility	1.87
Performance incentives for the facility	1.12
Performance incentives for health workers	1.00
Focus on performance and results	0.87
Increased capacity for planning and financial management	0.87
More autonomy	0.80
More active health facility governing committees	0.69
Higher pay for health workers (income effect)	0.57
More support from district authorities	0.50

We also note that ‘more funds available to the health facility’ was considered the aspect of RBF that contributed most to improved service delivery, 87% more important than ‘performance incentives for health workers’. ‘Performance incentives for the facility’ were also considered more important than performance incentives for health workers. Thus, the aspects of RBF considered most important for improving service delivery were also present in the DHFF scheme, albeit to a lesser extent.

Health workers also indicated that much of the gains of RBF could have been achieved without personal incentives. During phone interviews, health workers were asked what would happen to service quality if all performance payments went to the health facility and not to health workers (like in the DHFF programme). Despite strong incentives to emphasize the importance of personal bonuses, 50% of health workers in the intervention districts and 76% in the comparison districts responded that service quality would be equally good or better if all payments went to the health facility (‘phone survey, round 3').

## Discussion

Between 2016 and 2020, there was a general improvement in service coverage and quality in both intervention and comparison districts. Beyond this improvement, RBF led to statistically significant gains in 14 of the 24 incentivized items. RBF also improved 22 outcomes that were not directly incentivized—including BCG vaccine coverage, content of delivery care, communication, and responsiveness—indicating that RBF may contribute to broader health system strengthening.

These results are in line with other studies of RBF in the sense that (i) RBF had positive impacts on some, but not all, incentivized services, (ii) RBF had positive effects on some nonincentivized services, and (iii) RBF had few or no negative effects on both incentivized and nonincentivized services (Diaconu et al. 2021). An important contribution of this paper is that we show that these conclusions also may hold in a context where less comprehensive direct financing mechanisms are being implemented and there are strong, positive underlying trends in outcome variables. The magnitudes of the measured impacts, both on service utilization and service quality, are large compared to most other studies of RBF. Our findings contrast with those of de Walque et al. (2022), who pooled data from five countries (Zambia, Zimbabwe, Cameroon, Nigeria, and Rwanda) and found limited evidence of additional impacts of PBF beyond direct financing mechanisms.

RBF may have caused significant health benefits. For instance, the increase in the share of women who had their first ANC consultation before 12 weeks of gestation (10.7 pp) is an important improvement as early ANC allows for screening, test, and treatments that are most effective early in the pregnancy (e.g. provision of iron and folic acid supplements and treatments of sexually transmitted diseases) (Moller et al. 2017). Improved quality of ANC consultations may also have had important health benefits. First, blood samples were taken more frequently, which is important for diagnosing iron deficiency and identifying blood type and rhesus factor for improved follow-up. Second, more women were prescribed with iron or folic acid, which not only prevents maternal anaemia but also puerperal sepsis, preterm birth, and low birth weight (WHO 2016). Third, the increase in measurement of blood pressure and urine examination are important for diagnosing preeclampsia, which constitute a sizable risk for maternal and perinatal morbidity and mortality (Souza et al. 2013). Fourth, increased provision of intermittent preventive treatment of malaria may reduce the adverse effects of malaria on both maternal and foetal outcomes, including placental infection, clinical malaria, maternal anaemia, foetal anaemia, low birth weight, and neonatal mortality (WHO 2014). Finally, increased vaccination coverage for measles and BCG may also have caused important health benefits for infants.

Furthermore, RBF had positive effects for clients through more responsive and respectful treatment. Health workers listened more carefully, avoided harsh words to a greater extent, and provided more equitable services for rich and poor, and waiting times were reduced. We note in particular that RBF led to more respectful maternity care. This is important as the mistreatment of women in health facilities during childbirth is extensive (Bohren et al. 2019), and there is a great need for giving women a more positive childbirth experience (WHO 2018).

Despite improving women’s experiences with delivery services, RBF did not increase utilization of these services. This was surprising for the several reasons: (i) the pilot P4P programme implemented in another region in Tanzania from 2011 onwards had such effects, even though the baseline service utilization was considerably higher (Borghi et al. 2021), (ii) studies in other countries have shown that RBF tends to have stronger impact on institutional deliveries than on the coverage of other services (Neelsena et al. 2021), and (iii) the RBF programme in Tanzania had particularly strong incentives for increasing institutional deliveries as CHWs were incentivized for escorting pregnant women to health facilities for delivery. However, while the data suggest that CHWs escorted far more women than before, they apparently escorted women who would have delivered at a health facility anyway. It is also conceivable that the strong underlying positive trend in institutional deliveries (21.5 pp) reduced the potential of RBF to make further improvements.

Strong, positive underlying trends may also have reduced the potential positive effects of RBF for other services. We note, for instance, that vitamin A supplementation increased by 39.0 pp and mebendazole for children by 27.9 pp.

We measured only one statistically significant negative effect of RBF: a reduction in the use of family planning methods. This result is driven by a large increase in the use of implants in the comparison districts. For other family planning methods, there are no statistically significant effects (results not shown). We cannot rule out that this result may be driven by a campaign in the comparison districts to promote these specific products.

Regarding the mechanisms that contributed to change, we find few significant effects on our intermediary outcomes, apart from an increase in health worker job satisfaction. This finding is consistent with data suggesting that (i) more resources were a main driver for the results of RBF, (ii) a significant share of these resources was spent on improving working conditions for health workers, and (iii) improved physical conditions of the health facility were a main driver for improved job satisfaction. A positive effect of RBF on job satisfaction but no effect on ‘personal drive’ is consistent with findings from Malawi (Lohmann et al. 2018). Broadly similar findings are reported by Lamba et al. (2025) from a study covering six countries. These studies also suggest that improvement in the working environment was a key factor for this change.

Improved job satisfaction may also explain improvements in content of care and process quality, in particular those aspects that were not directly incentivized, did not pay off indirectly in terms of improved service utilization (e.g. delivery services), and did not require any physical equipment.

Unfortunately, we do not have access to data that would allow us to assess the costs at which these gains were achieved. However, it is not evident that a full-scale RBF scheme is necessary to realize such improvements. Suggestive evidence on the mechanisms of change indicates that the key drivers of RBF’s impact were increased resources and stronger incentives at the facility level—both already embedded in the DHFF scheme. Further strengthening the DHFF approach is therefore a viable and potentially more cost-efficient alternative. While the RBF programme was implemented in nine regions—including Mwanza—with relatively poor economic and health indicators, the differences compared with other regions were not very large. We therefore expect the results to relevant beyond the study area. For instance, Mwanza typically performed 5–10 pp below the national average on indicators, such as institutional deliveries, child vaccinations, and unmet need for family planning.

### Robustness

We reanalysed the data with standard errors clustered at the district level using wild bootstrapping (Roodman et al. 2019). Confidence intervals widened for about two-thirds of the outcomes and narrowed for others. The number of outcome variables showing a statistically significant increase at the 5% level dropped from 36 to 26. At the 10% level, however, there were still 39 positive effects, compared to 41 with clustering at the facility level (Supplementary Tables: District level clustering).

### Limitations

We cannot rule out that unobserved time-variant factors may have influenced results differently in the intervention and comparison districts. We are aware that donor-funded programmes were implemented in districts in Mwanza region to increase CHWs’ provision of nutritional services, but this is unlikely to have a substantive effect on our results. We are not aware of any other such factors.

Since RBF implementation had partially begun in the comparison districts by the time of the endline survey, we cannot rule out its possible effect on some of the outcomes measured at the health facility level. (At the household level, however, we only interviewed women who had delivered before RBF was implemented.) The promise of RBF funds might have affected health worker motivation and efforts to improve performance and biased some of our estimates. Such biases are most likely for estimated effects on intermediary outcomes, such as health workers’ efforts to attract more patients, which might be downward biased.

Our sample included households residing within 5 km of a health facility, which likely includes around 70% of the population (McIntyre et al. 2008). It is conceivable that the effects of RBF would differ for more remote households, particularly regarding service utilization. In theory, these effects could be either larger or smaller.

The sample of health workers at endline differed from the baseline sample on some observable characteristics (Supplementary Table S9). Although our analysis accounted for differences in observable characteristics, unobservable differences might have affected estimates of some of our intermediary outcomes.

## Conclusions

The implementation of DHFF alone in Tanzania was insufficient to fully realize the potential effects of health financing reforms on service delivery. Combining RBF with DHFF led to additional, substantial improvements.

It would be premature, however, to conclude that RBF should be layered on top of other direct facility financing approaches. Further gains in service delivery may be achieved more simply and cost-effectively by further developing the DHFF scheme rather than implementing a comprehensive RBF programme alongside it.

## Supplementary Material

czag058_Supplementary_Data

## Data Availability

Data can be made available upon request, pending approval by the Institutional Review Board (IRB) of the authors’ institution. For data requests, please contact the IRB secretary.

## Reflexivity statement

The authors include one female and four males and span multiple levels of seniority. While all authors specialize in economic and social development in the Global South, three specialize in health systems research, one in epidemiology, and one in development economics more broadly. Three of the authors have extensive experience in conducting field work in Tanzania.
