# Supplementary material for: Does dosage matter? Effects of results-based financing layered on top of less comprehensive direct facility financing in Tanzania
Source: Health Policy Plan. 2026 May 13;41(6):988–1003. doi: 10.1093/heapol/czag058 (PMC13276258; doi:10.1093/heapol/czag058)
Supplement: czag058_Supplementary_Data [file czag058_supplementary_data.zip › Supplementary Tables S1-S13.docx]

**Supplementary Tables S1-S13.**

**Table S1: Incentivized services, dispensaries and health centres.**

|  | **Fee per unit (USD)** |
| --- | --- |
| Outpatient consultations | 0.25 |
| TASAF beneficiaries seeking outpatient care | 0.75 |
| First ANC visit with gestation age <12 weeks | 5.00 |
| At least 4 ANC visits | 3.75 |
| Pregnant women receiving two doses of malaria prevention (IPT2) | 0.75 |
| HIV positive pregnant women receiving ARVs | 2.00 |
| Institutional deliveries | 12.50 |
| Postnatal services within 3-7 days after delivery | 5.00 |
| Children under one year immunized against measles | 1.00 |
| Children under five receiving Vit. A supplements | 0.50 |
| New users of modern family planning methods | 3.50 |
| Clients initiated to counsel and test for HIV | 0.37 |
| HIV exposed infants receiving ARVs | 3.00 |
| TB suspected clients referred after being screened | 5.00 |

Source: *MoHSW, 2015.*

**Table S2: Groups of quality indicators, dispensaries.***

|  | **Max score** |
| --- | --- |
| Hygiene and sanitation | 9 |
| Privacy | 4 |
| Water supply | 4 |
| Waste management | 6 |
| ANC (incl. content of care) | 13 |
| Labour ward (structural quality) | 13 |
| Postnatal care (incl. content of care) | 9 |
| Maternal death audits | 10 |
| Perinatal death audits | 10 |
| Family planning (structural quality and content of care) | 17 |
| Immunization (structural quality) | 7 |
| Nutrition for under-five children (content of care) | 4 |
| Pharmacy (drugs in stock) | 9 |
| Community (involvement in RBF management) | 5 |
| Community health fund (enrollment increase) | 10 |
| Facility profile reports available | 6 |
| Transparency (information provided on notice board) | 5 |
| Client satisfaction | 20 |

**A somewhat more comprehensive checklist was used at health centres and hospitals (MoHSW 2015).*

**Table S3: Incentivized services, Community Health Workers.**

|  | **Fee per unit (USD)** |
| --- | --- |
| Number of households visits | 0.75 |
| Number of women escorted to health facilities for delivery | 5.00 |
| Number of non-institutional maternal and perinatal deaths reported | 2.50 |

**Table S4**. **Items used to measure satisfaction with working conditions (mean (sd)).**

|  | **Comparison** | | **Intervention** | | **DID (%)^#^** |
| --- | --- | --- | --- | --- | --- |
|  | **Baseline** | **Endline** | **Baseline** | **Endline** |  |
| **Retained items^a^** |  |  |  |  |  |
| Availability of medicines and supplies | 2.46 (1.32) | 3.34 (1.24)* | 2.65 (1.3) | 3.84(1.27)* | 11.7 |
| Availability of functioning equipment | 2.27 (1.14) | 3.42 (1.26)* | 2.5 (1.27) | 3.97(1.11)* | 12.8 |
| The physical condition of the health facility | 2.69 (1.39) | 3.17 (1.42)* | 2.72 (1.38) | 3.88(1.26)* | 25.0 |
| Opportunity to be rewarded for hard work, financially or otherwise | 2.49 (1.39) | 2.79 (1.47) | 2.58 (1.41) | 3.1(1.51)* | 8.5 |
| Safety and security in the community | 3.33 (1.47) | 3.46 (1.47) | 3.54 (1.47) | 3.89(1.39)* | 6.2 |
| **Other items** |  |  |  |  |  |
| Working relationships with the district | 3.91 (1.24) | 4.33 (0.92)* | 3.68 (1.42) | 4.37(1.02)* |  |
| Overall quality of the management of the facility | 3.5 (1.28) | 3.95 (1.01)* | 3.46 (1.35) | 4.34(0.99)* |  |
| Opportunities to upgrade your skills and knowledge through training | 3.15 (1.48) | 3.42 (1.33) | 3.22 (1.47) | 3.6(1.32)* |  |
| Ability to provide high quality of care | 3.44 (1.3) | 4.08 (1.08)* | 3.45 (1.32) | 4.08(1.12)* |  |
| Opportunities for promotion | 2.2 (1.32) | 2.4 (1.46) | 2.61 (1.54) | 2.34(1.55) |  |
| Living accommodation | 2.03 (1.32) | 2.62 (1.55)* | 2.23 (1.42) | 2.73(1.57)* |  |

*p<0.05 (baseline-endline comparison).

^#^ Difference-in-difference per item calculated without any controls for illustrative purposes.

^a^ Goodness of fit: χ^2^_(5)_ =8.093 (p=0.151), root mean squared error of approximation (RMSEA) = 0.033, comparative fit index (CFI) = 0.993, and standardized root mean squared residual (SRMR) = 0.023.

**Table S5**. **Items used to measure personal drive (mean (sd)).**

|  | **Comparison** | | **Intervention** | |
| --- | --- | --- | --- | --- |
|  | **Baseline** | **Endline** | **Baseline** | **Endline** |
| **Retained items^a^** |  |  |  |  |
| I am proud of the work I do at this facility | 2.96 (0.90) | 3.46 (0.68)* | 2.94 (0.78) | 3.43 (0.67)* |
| I am keenly aware of the income goals I have for myself | 3.46 (0.68) | 3.41 (0.62) | 3.34 (0.63) | 3.5 (0.58)* |
| I enjoy trying to solve the problems that patients come with | 3.6 (0.51) | 3.64 (0.48) | 3.5 (0.54) | 3.66 (0.54)* |
| I feel that the greatest reward with this job is that I can help other people | 3.34 (0.79) | 3.58 (0.55)* | 3.28 (0.73) | 3.5 (0.61)* |
| I am keenly aware of the career goals I have set for myself | 3.67 (0.47) | 3.65 (0.48) | 3.56 (0.53) | 3.66 (0.49) |
| I have to feel that I get something in return for the work that I do | 2.91 (1.06) | 2.95 (1.04) | 2.99 (0.96) | 2.88 (1.06) |
| **Other items** |  |  |  |  |
| I prefer having someone set clear goals for me in my work | 3.0 (0.99) | 2.61 (1.14)* | 2.66 (1.03) | 2.68 (1.17) |
| I am strongly motivated by the recognition I get from other people | 3.30 (0.73) | 3.44 (0.65) | 3.23 (0.7) | 3.5 (0.61)* |
| I am strongly motivated by the income I can earn at work | 2.49 (1.01) | 2.66 (1.01) | 2.46 (0.98) | 2.74 (1.08)* |
| I feel bad If I do not follow the professional guidelines for my work | 3.61 (0.59) | 3.5 (0.76) | 3.46 (0.62) | 3.54 (0.73) |
| It is important that my peers recognize my work as professional | 3.58 (0.69) | 3.6 (0.6) | 3.57 (0.55) | 3.55 (0.61) |
| To be motivating hard work must be rewarded with more status and money | 2.17 (1.21) | 1.96 (1.16) | 2.42 (1.14) | 1.95 (1.09)* |
| My job makes me feel good about myself | 3.60 (0.59) | 3.69 (0.56) | 3.58 (0.56) | 3.71 (0.51) |
| I'm less concerned with what work I do than what I get for it (reversed) | 3.83 (0.51) | 3.92 (0.34) | 3.72 (0.62) | 3.91 (0.42)* |
| I feel it is not so important doing a good job if nobody else knows about it | 1.47 (0.84) | 1.46 (0.89) | 1.53 (0.82) | 1.68 (1.08) |
| As long as I can do what I enjoy, I'm not that concerned about exactly what income or awards I earn | 2.77 (1.02) | 3.19 (1.0)* | 2.89 (0.98) | 3.08 (1.03) |

* p<0.05 (baseline-endline comparison).

^a^ Goodness of fit: χ^2^_(9)_=13.538 (p=0.140), RMSEA=0.030, CFI=0.992, and SRMR=0.025..

**Table S6. Step-by-step testing of measurement invariance of satisfaction with working conditions between comparison and intervention groups.**

| **Model** | **Invariance** | **Model**  **compared** | **χ^2^(df)** | **Δχ^2^** | **p value** | **RMSEA** | **CFI** | **TIL** | **SRMR** | **CD** | **Decision** |
| --- | --- | --- | --- | --- | --- | --- | --- | --- | --- | --- | --- |
| M1 | Configural |  | 17.48(10) |  |  | 0.051 | 0.983 | 0.967 | 0.035 | 0.801 |  |
| M2 | Metric | M1 | 20.55(14) | 3.07(4) | 0.546 | 0.041 | 0.986 | 0.979 | 0.042 | 0.793 | Accept |
| M3 | Scalar | M2 | 23.3(18) | 2.75(4) | 0.601 | 0.032 | 0.988 | 0.987 | 0.042 | 0.789 | Accept |

*Notes:*

N=568 (283 in intervention and 285 in comparison).

Configural invariance: No constraints.

Metric invariance: Loadings constrained.

Scalar invariance: Loadings and intercepts constrained and if achieved, group mean comparison is possible.

RMSEA=Root mean squared error of approximation; CFI=Comparative fit index; TIL**=**Tucker-Lewis index; SRMR=Standardized root mean squared residual; CD=Coefficient of determination.

**Table S7. Step-by-step testing of measurement invariance of “personal drive” between comparison and intervention groups.**

| **Model** | **Invariance** | **Model**  **compared** | **χ^2^(df)** | **Δχ^2^** | **p value** | **RMSEA** | **CFI** | **TIL** | **SRMR** | **CD** | **Decision** |
| --- | --- | --- | --- | --- | --- | --- | --- | --- | --- | --- | --- |
| M1 | Configural |  | 24.25(18) |  |  | 0.035 | 0.990 | 0.983 | 0.033 | 0.789 |  |
| M2 | Metric | M1 | 27.97(23) | 3.72(5) | 0.59 | 0.028 | 0.992 | 0.989 | 0.039 | 0.787 | Accept |
| M3 | Scalar | M2 | 29.1(28) | 1.13(5) | 0.95 | 0.012 | 0.998 | 0.998 | 0.039 | 0.787 | Accept |

*Notes: Same as Table 6.*

**Table S8. Characteristics of mothers interviewed (%, unless specified).**

|  | **Control** | | **Intervention** | |
| --- | --- | --- | --- | --- |
|  | **Baseline** | **Endline** | **Baseline** | **Endline** |
| N | 1,499 | 1,875 | 1,499 | 1,870 |
| Wealth score (mean, sd) | -0.227  (1.098) | 0.140  (0.986) | -0.142  (0.982) | 0.155  (0.889) |
| Number of alive children (mean, sd) | 3.3  (2.1) | 3.4  (2.1) | 3.6  (2.4) | 3.7  (2.3) |
| **Highest level of schooling** |  |  |  |  |
| Less than standard seven | 10.9 | 11.7 | 17.2 | 16.3 |
| Standard seven | 76.1 | 72.3 | 69.0 | 69.9 |
| Form 4 and above | 13.0 | 16.0 | 13.8 | 13.8 |
| **Main current occupation** |  |  |  |  |
| Public servant | 0.9 | 1.0 | 1.3 | 0.4 |
| Private formal sector | 0.2 | 0.2 | 0.1 | 0.1 |
| Subsistence farmer | 76.0 | 71.8 | 75.3 | 73.9 |
| Large scale farmer | 0.2 | 1.2 | 0.3 | 2.2 |
| Self-employed/small business | 11.5 | 14.2 | 9.5 | 12.4 |
| Self-employed / large business | 0.3 | 0.0 | 0.1 | 0.1 |
| Taking care of the home/children | 9.6 | 10.7 | 12.1 | 9.9 |
| Student | 0.1 | 0.1 | 0.2 | 0.1 |
| Looking for work | 0.7 | 0.7 | 0.5 | 0.6 |
| Other | 0.7 | 0.2 | 0.6 | 0.4 |
| **Marital status** |  |  |  |  |
| Never married | 11.0 | 9.5 | 11.6 | 10.9 |
| Married | 60.2 | 59.0 | 55.0 | 54.8 |
| Living with partner | 23.2 | 23.6 | 27.0 | 27.1 |
| Divorced/separated | 3.2 | 4.9 | 5.2 | 6.3 |
| Widowed | 2.3 | 3.0 | 1.1 | 0.9 |
| Other | 0.0 | 0.0 | 0.1 | 0.2 |

**Table S9. Characteristics of health workers interviewed at baseline and endline (%, unless specified).**

|  | **Control** | | **Intervention** | |
| --- | --- | --- | --- | --- |
|  | **Baseline** | **Endline** | **Baseline** | **Endline** |
| N | 136 | 149 | 137 | 146 |
| Gender: Female | 44.1 | 34.9 | 40.1 | 29.5 |
| Age (mean, sd) | 35.3  (11.2) | 36.2  (10.1) | 38.5  (11.7) | 37.7  (10.2) |
| Grew up in the district | 21.3 | 21.5 | 16.8 | 17.1 |
| Years served in facility  (mean, sd) | 4.3  (5.9) | 6.0  (6.9) | 5.3  (6.8) | 6.3  (5.9) |
| Years served in health sector  (mean, sd) | 10.8  (12.0) | 11.6  (11.5) | 12.6  (12.9) | 11.8  (10.6) |
| In-charge of the facility | 37.5 | 15.4 | 37.2 | 15.1 |
| **Health/medical training** |  |  |  |  |
| Medical officer | 5.9 | 4.7 | 16.1 | 7.5 |
| Ass. Medical officer | 11.7 | 2.0 | 7.3 | 1.4 |
| Clinical officer | 11.7 | 7.4 | 11.0 | 7.5 |
| Enrolled nurse | 12.5 | 9.4 | 5.8 | 13.7 |
| Registered nurse | 11.8 | 18.1 | 13.9 | 19.2 |
| Nurse midwife | 24.3 | 21.5 | 24.1 | 19.2 |
| Medical attendant/nurse assistant | 19.9 | 30.9 | 20.4 | 28.1 |
| Other | 2.1 | 6.0 | 1.5 | 3.4 |

**Table S10. Characteristics of Community Health Workers interviewed at baseline and endline (%, unless specified).**

|  | **Control** | | **Intervention** | |
| --- | --- | --- | --- | --- |
|  | **Baseline** | **Endline** | **Baseline** | **Endline** |
| N | 104 | 147 | 103 | 147 |
| Age (mean, sd) | 46.5  (8.4) | 43.4  (16.0) | 44.9  (8.5) | 44.1  (13.1) |
| Years working as a CHW (mean, sd) | 17.2  (9.3) | 16.3  (10.0) | 14.2  (7.9) | 14.5  (8.8) |
| Gender: Female | 38.5 | 53.7 | 40.8 | 48.3 |
| Grew up in the district | 76.0 | 71.4 | 69.9 | 70.1 |
| **Health/medical training** | | | | |
| No training | 4.8 | 10.9 | 5.8 | 15.6 |
| Less than three months | 58.7 | 67.3 | 53.4 | 53.1 |
| Between 3 and 6 months | 21.2 | 12.2 | 23.3 | 13.6 |
| Between 6 and 12 months | 7.7 | 4.1 | 9.7 | 5.4 |
| More than 12 months | 7.7 | 5.4 | 7.8 | 12.2 |

**Table S11. Adjusting for multiple testing.**

| **Category** | **Dependent variable** | **p-value** | **Sharpened q-value** |
| --- | --- | --- | --- |
| Service utilization | ANC consultation before 12 weeks | .000296 | .002 |
|  | At least 4 ANC visits | .005938 | .017 |
|  | Institutional delivery | .915584 | .432 |
|  | Postnatal check-up within 3-7 days | .418447 | .355 |
|  | Measles vaccination | .01 | .021 |
|  | Vitamin A supplementation | .733 | .432 |
|  | BCG vaccination | .000013 | .001 |
|  | DPTHibHepB3 vaccination | .211 | .178 |
|  | Mebendazole | .164589 | .159 |
|  | Using any family planning method | .044923 | .057^§^ |
| Content of care | Measured height | .142596 | .11 |
|  | Took blood sample | .002694 | .012 |
|  | Measured blood pressure | .031843 | .039 |
|  | Listened to the baby’s heart | .001766 | .011 |
|  | Gave or prescribed iron or folic acid | .000384 | .006 |
|  | Gave IPT, at least two doses | .036885 | .039 |
|  | Gave tetanus vaccination | .865323 | .351 |
|  | Gave Mebendazole | .09744 | .079 |
|  | Measured weight | .337487 | .203 |
|  | Analysed urine | .016578 | .029 |
|  | Gave voucher for bednet | .849768 | .351 |
|  | Checked blood pressure | .012327 | .028 |
|  | Took blood test | .01492 | .029 |
|  | Asked about abnormal bleeding | .173683 | .126 |
|  | Examined abdomen | .000541 | .006 |
|  | Examined breasts | .008991 | .023 |
|  | Examined vagina | .005417 | .018 |
|  | Weighed infant at birth | .827793 | .351 |
|  | Breastfeeding within an hour | .323569 | .203 |
|  | Vaccinated the newborn | .8213 | .351 |
| Structural quality | Drugs available (23 items) | .008 | .022 |
|  | Functional medical equipment (17 items) | .228 | .19 |
|  | Medical supplies (9 items) | .16 | .143 |
|  | Contraceptive supplies (8 items) | .006 | .022 |
|  | Electricity supply | 0.537 | .261 |
|  | Improved water source (piped, well, pump) | 0.094 | .118 |
|  | Functioning toilet (VIP or flush) | 0.105 | .118 |
|  | The facility was not dirty | .009748 | .022 |
|  | The delivery room was clean | .055567 | .081 |
|  | The drugs the mother needed were available | .511156 | .261 |
|  | The hours the facility is open were adequate | .000144 | .001 |
|  | Drugs available (last visit) | 2.536e-06 | .001 |
|  | Facility found open (any visit during past 2 years) | .345 | .206 |
| Process quality: Communication | Discussed and advised on the place of delivery | .485505 | .263 |
|  | Staff introduced themselves | .003818 | .035 |
|  | Asked if the mother wanted someone to support her during delivery | .040083 | .068^§^ |
|  | Explained what they were doing before conducting any procedure | .055415 | .083 |
|  | Advised what to do to make the mother more comfortable during pain | .004444 | .035 |
|  | Did a good job at explaining the progress of the delivery | .626341 | .335 |
|  | Discussed family planning | .354177 | .216 |
|  | Talked about danger signs | .039653 | .068^§^ |
|  | Told the mother when to come back | .011636 | .045 |
|  | Gave advice about breastfeeding | .440308 | .256 |
|  | Discussed signs of newborn complications | .013115 | .045 |
|  | Explained how FP methods work | .274607 | .185 |
|  | Explained the advantages and disadvantages of a particular method | .09364 | .094 |
|  | When the method of choice was not available, health worker told where she could receive it | .269587 | .185 |
|  | Explained what to do in case of side effects | .027334 | .064^§^ |
| Process quality: Responsiveness | Time spent with health provider during the delivery was not too low | .672155 | .176 |
|  | Staff helped make the mother more comfortable during labour | .005303 | .01 |
|  | Staff came to assist the mother when she called for help | .918657 | .226 |
|  | Privacy was sufficiently respected | .001793 | .006 |
|  | Was treated with respect and dignity | .081051 | .046 |
|  | Staff’s kindness | .008 | .012 |
|  | Staff took time to listen to carefully | .000452 | .005 |
|  | No harsh words to the patients | .026 | .023 |
|  | Treatment provided equally to rich and the poor | .038 | .028 |
|  | Waiting for less than an hour (last visit) | .001645 | .006 |
| Patient satisfaction | The overall quality of the service was satisfactory | .076896 | .084 |
|  | Mother would recommend the facility to friends | .220326 | .131 |
|  | Satisfied with the overall quality of the service | .002274 | .007 |
| Mechanisms (intermediary outcomes) | Satisfaction with working condition | .008 | .034 |
|  | Personal drive | .51 | .682 |
|  | Staff present at health facility | 0.187 | .453 |
|  | Number of HFGC meetings in the past 12 months | 0.162 | .453 |
|  | Met with supervisor during the past 90 days | 0.225 | .474 |
|  | Received feedback on the quality of work from external supervisor during the last meeting | 0.595 | .682 |
|  | Days a week open for outpatient services | 0.534 | .682 |
|  | Facility offers 24hours delivery services | 0.981 | .828 |
|  | Facility conducts outreaches services | 0.492 | .682 |
|  | Did not pay for delivery care services | .000347 | .005 |
|  | Did not purchase supplies to bring for the birth | .135 | .453 |
|  | Number of women escorted last 3 months | .005 | .031 |
|  | Number of households visited last week | .849 | .738 |

^§^ p-value<0.05 and sharpened q-value>0.05.

**Table S12. Strategies used to increase service utilization (%).**

|  | **Control** | | **Intervention** | | **DID estimation (95% CI)** | | | |
| --- | --- | --- | --- | --- | --- | --- | --- | --- |
|  | **Base** | **End** | **Base** | **End** | **Estimate** | Lower | Upper | **p value** |
| **Delivery service** |  |  |  |  |  |  |  |  |
| Encourage CHWs or traditional birth assistants to bring women to the facility | 61.0 | 81.9* | 62.0 | 92.5* | 9.8 | -4.2 | 23.7 | 0.169 |
| Provide more prompt attention to women who deliver | 19.1 | 42.3* | 26.3 | 48.6* | -1.5 | -16.6 | 13.7 | 0.850 |
| Provide gifts to mothers (soap, Khanga, bednets, sweets) | 8.1 | 34.9* | 12.4 | 43.2* | 3.4 | -10 | 16.8 | 0.620 |
| Increase opening hours | 10.3 | 32.2* | 21.9 | 43.2* | -1.1 | -15.1 | 13 | 0.881 |
| Ensure that the facility is better equipped with necessary supplies for deliveries | 13.2 | 36.2* | 21.2 | 50.0* | 5.1 | -9.4 | 19.6 | 0.490 |
| Made maternity home available | 6.2 | 30.9* | 19.7 | 38.4* | -6.5 | -20 | 7 | 0.344 |
| Improve transport opportunities for pregnant women | 2.2 | 15.4* | 10.2 | 18.5* | -5.9 | -16.3 | 4.6 | 0.270 |
| Urge women to deliver at facility | 70.6 | 84.6* | 64.2 | 84.3* | 3.9 | -10 | 17.8 | 0.580 |
| Lower/reduce charges | 2.2 | 12.1* | 5.1 | 13.7* | -1.5 | -10.4 | 7.5 | 0.751 |
| Distribute free delivery kits | 20.6 | 29.5 | 15.3 | 41.8* | 16.9 | 2.5 | 31.2 | 0.022 |
| **Outpatient service** |  |  |  |  |  |  |  |  |
| Increase opening hours | 5.9 | 34.9* | 11.0 | 45.2* | 4.8 | -8.3 | 17.8 | 0.474 |
| Health workers being less absent than before | 7.4 | 30.9* | 13.1 | 42.5* | 5.8 | -7.2 | 18.9 | 0.381 |
| Health workers being more friendly to patients than before | 16.2 | 47.0* | 31.4 | 55.5* | -7.9 | -23.2 | 7.4 | 0.310 |
| Urge people to come to the facility | 61.0 | 68.5 | 65.7 | 67.8 | -4.8 | -20.6 | 11 | 0.552 |
| Urge community health workers to send patients to the facility | 32.4 | 41.6 | 36.5 | 58.9* | 12.4 | -3.7 | 28.5 | 0.131 |
| Ensure less stock out of drugs | 20.6 | 55.0* | 32.1 | 66.4* | -1.8 | -17.1 | 13.5 | 0.817 |
| Ensure that the facility is better equipped for outpatient consultations | 10.3 | 34.2* | 21.2 | 46.6* | 0.4 | -13.7 | 14.6 | 0.952 |
| Reduce user charges | 6.6 | 16.8* | 11.7 | 20.6* | -1.2 | -12.5 | 10.1 | 0.831 |

^¤^Adjusted for years served in the facility and in a health sector, growing up in the district, being in charge of the facility, medical/health training, age and sex of the health workers.

*Significant at 0.05 level (baseline-endline comparison and DID estimation).

**Table S13. Staff composition based on roster (%, unless specified).**

|  | **Control** | | **Intervention** | | **DID estimation** | | | |
| --- | --- | --- | --- | --- | --- | --- | --- | --- |
|  | **Base** | **End** | **Base** | **End** | **Estimate** | Lower | Upper | **p value** |
| **Demography** |  |  |  |  |  |  |  |  |
| Sex: Female | 60.6 | 51.2* | 70.9 | 67.6 | 6.2 | -3.2 | 15.6 | 0.197 |
| Age (mean (sd)) | 36.5  (11.5) | 35.8  (10.4) | 37.0  (10.7) | 37.8  (10.1) | 1.5 | -0.6 | 3.6 | 0.155 |
| **Position** |  |  |  |  |  |  |  |  |
| Medical/clinical/assistant officers | 12.4 | 24.0* | 18.6 | 23.7 | -6.5 | -14.3 | 1.2 | 0.098 |
| Nurse/midwife | 6.3 | 34.6* | 10.8 | 40.6* | 1.5 | -6.2 | 9.2 | 0.705 |
| Nurse assistant/medical attendant | 42.4 | 28.2* | 39.1 | 25.3* | 0.4 | -8.8 | 9.7 | 0.923 |

*Significant at 0.05 level (baseline-endline comparison and DID estimation).
