# Supplementary material for: Does dosage matter? Effects of results-based financing layered on top of less comprehensive direct facility financing in Tanzania
Source: Health Policy Plan. 2026 May 13;41(6):988–1003. doi: 10.1093/heapol/czag058 (PMC13276258; doi:10.1093/heapol/czag058)
Supplement: czag058_Supplementary_Data [file czag058_supplementary_data.zip › Supplementary Tables. District level clustering.docx]

**Supplementary Tables: Results with District-Level Wild Bootstrap Standard Errors.**

Reports those tables that included statistically significant effects in the main text.

**Table D2. Effects of RBF on aggregate service delivery indicator indices**^#^ **(%).**

|  | **Comparison** | | **Intervention** | | **DID estimation (95% CI)** | | | |
| --- | --- | --- | --- | --- | --- | --- | --- | --- |
|  | **Base** | **End** | **Base** | **End** | **Estimate** | Lower | Upper | **p value** |
| Service utilization (10 items) | 41.5 | 62.8* | 40.9 | 65.0* | **3.3** | -1.5 | 8.4 | 0.184 |
| Content of care (20 items) | 57.0 | 70.0* | 57.4 | 76.2* | **5.7*** | 1.6 | 9.5 | 0.013 |
| Structural quality |  |  |  |  |  |  |  |  |
| *Enumerators’ observation (7 items^¤^)* | 56.0 | 76.1* | 54.1 | 83.7* | **9.3*** | 0.4 | 17.7 | 0.042 |
| *Mothers’ observation (6 items)* | 67.7 | 74.9* | 70.0 | 83.2* | **6.8** | -0.6 | 12.5 | 0.071 |
| Process quality (25 items) | 62.3 | 68.5* | 59.3 | 71.7* | **6.2*** | 2.8 | 9.9 | 0.003 |
| Client satisfaction (3 items) | 72.0 | 82.0* | 76.2 | 90.4* | **5.1** | -0.5 | 14.4 | 0.294 |

^#^Indexes are mean value of responses, in percent. All responses are coded on a scale from 0 to 1.

*Significant at 0.05 level (baseline-endline comparison and DID estimation).

^¤^Some items are indexes of underlying variables; 60 underlying variables are captured in total.

**Table D3. Effects on service utilization (%).**

|  | **Comparison** | | **Intervention** | | **DID estimation (95% CI)** | | | |
| --- | --- | --- | --- | --- | --- | --- | --- | --- |
|  | **Base** | **End** | **Base** | **End** | **Estimate** | Lower | Upper | **p value** |
| **Antenatal** |  |  |  |  |  |  |  |  |
| ^@^ANC consultation before 12 weeks | 21.1 | 30.8* | 17.1 | 36.1* | **10.6*** | 1.2 | 19.3 | 0.034 |
| ^@^At least 4 ANC visits | 60.9 | 78.7* | 52.2 | 78.1* | **7.7*** | 0.4 | 15.5 | 0.039 |
| **Delivery and postnatal** |  |  |  |  |  |  |  |  |
| ^@^Institutional delivery | 54.7 | 76.2* | 68.4 | 89.4* | **-0.3** | -8.7 | 9.0 | 0.946 |
| ^@^Postnatal check-up within 3-7 days | 65.9 | 73.4* | 69.8 | 82.1* | **4.6** | -6.6 | 21.6 | 0.582 |
| **Child immunization and nutrition supplementation** | | | | |  |  |  |  |
| ^@^Measles vaccination | 68.0 | 82.9* | 62.9 | 84.2* | **8.7** | -1.0 | 20.2 | 0.078 |
| ^@^Vitamin A supplementation | 19.1 | 58.1* | 20.8 | 59.2* | **1.3** | -8.8 | 12.6 | 0.808 |
| BCG vaccination | 92.2 | 98.3* | 80.9 | 98.1* | **11.8*** | 0.7 | 23.2 | 0.033 |
| DPTHibHepB3 vaccination | 72.2 | 88.7* | 72.7 | 91.8* | **3.6** | -8.7 | 16.7 | 0.541 |
| Mebendazole | 14.1 | 42.0* | 18.0 | 41.7* | **-3.6** | -7.5 | 0.4 | 0.075 |
| **Family planning** |  |  |  |  |  |  |  |  |
| ^@^Using any family planning method | 18.4 | 32.8* | 15.8 | 25.8* | **-4.3*** | -7.9 | -0.3 | 0.034 |

^@^Directly incentivized service.

*Significant at 0.05 level (baseline-endline comparison and DID estimation).

**Table D4. Effects on content of care (%).**

|  | **Comparison** | | **Intervention** | | **DID estimation (95% CI)** | | | |
| --- | --- | --- | --- | --- | --- | --- | --- | --- |
|  | **Base** | **End** | **Base** | **End** | **Estimate** | Lower | Upper | **p value** |
| **Antenatal care** |  |  |  |  |  |  |  |  |
| ^@^Measured height | 62.7 | 70.6* | 69.6 | 83.6* | **4.7** | -1.8 | 12.0 | 0.144 |
| ^@^Took blood sample | 84.8 | 93.1* | 79.9 | 95.6* | **7.0** | -2.6 | 16.7 | 0.137 |
| ^@^Measured blood pressure | 46.8 | 58.0* | 55.1 | 74.3* | **8.1** | -1.2 | 17.8 | 0.086 |
| ^@^Listened to baby’s heart | 97.5 | 98.0 | 95.5 | 99.0* | **3.3*** | 0.5 | 6.1 | 0.028 |
| ^@^Gave or prescribed iron or folic acid | 92.0 | 94.5* | 87.1 | 96.5* | **6.8*** | 0.4 | 13.8 | 0.035 |
| ^@^Gave IPT, at least two doses | 71.0 | 82.0* | 66.0 | 83.8* | **6.4** | -0.7 | 13.0 | 0.075 |
| ^@^Gave tetanus vaccination | 70.7 | 74.5* | 71.7 | 76.7* | **-0.4** | -4.9 | 4.5 | 0.851 |
| Gave Mebendazole | 46.1 | 63.6 * | 44.6 | 67.9* | **5.1** | -0.8 | 11.3 | 0.084 |
| Measured weight | 93.2 | 95.8* | 93.5 | 97.7* | **1.9** | -4.0 | 8.3 | 0.46 |
| Analysed urine | 31.2 | 39.3* | 34.1 | 51.6* | **8.6** | -2.5 | 19.6 | 0.114 |
| Gave voucher for bed net | 15.9 | 89.8* | 21.5 | 95.6* | **0.5** | -3.7 | 5.3 | 0.815 |
| **Delivery care – the mother** |  |  |  |  |  |  |  |  |
| Checked blood pressure | 19.0 | 36.4* | 23.7 | 50.6* | **9.5*** | 0.9 | 17.9 | 0.035 |
| Took blood test | 17.0 | 33.6* | 18.5 | 45.2* | **9.2*** | 2.1 | 17.2 | 0.01 |
| Asked about abnormal bleeding | 18.9 | 49.9* | 22.6 | 58.9* | **4.6** | -0.8 | 10.2 | 0.087 |
| Examined abdomen | 63.7 | 72.7* | 61.3 | 82.3* | **13.4*** | 8.8 | 19.3 | <0.001 |
| Examined breasts | 38.0 | 43.9* | 35.4 | 55.7* | **12.5*** | 6.2 | 19.3 | 0.001 |
| Examined vagina | 65.9 | 78.0* | 60.8 | 83.7* | **11.8*** | 5.7 | 18.4 | 0.002 |
| **Delivery care – the infant** |  |  |  |  |  |  |  |  |
| Weighed infant at birth | 94.3 | 95.5 | 97.0 | 98.3* | **-0.3** | -2.5 | 1.7 | 0.769 |
| Breastfeeding within an hour | 40.0 | 60.5* | 47.6 | 70.1* | **3.0** | -4.9 | 11.5 | 0.451 |
| Vaccinated the infant | 50.7 | 52.3 | 53.9 | 56.2 | **-0.9** | -11.1 | 8.4 | 0.862 |

^@^Directly incentivized service.
*Significant at 0.05 level (baseline-endline comparison and DID estimation).

**Table D5. Effects on structural quality (%).**

|  | **Comparison** | | **Intervention** | | **DID estimation (95% CI)** | | | |
| --- | --- | --- | --- | --- | --- | --- | --- | --- |
|  | **Base** | **End** | **Base** | **End** | **Estimate** | Lower | Upper | **p value** |
| **Health facility characteristics (as observed by enumerators)** | | | | | | | |  |
| ^@^Drugs available (23 items) | 57.6 | 65.2* | 58.3 | 75.6* | **9.7** | -3.6 | 22.5 | 0.141 |
| ^@^Functional medical equipment (17 items) | 59.1 | 80.6* | 59.0 | 84.2* | **3.7** | -5.9 | 15.1 | 0.548 |
| ^@^Medical supplies (9 items) | 53.6 | 72.3* | 53.6 | 77.2* | **4.9** | -6.2 | 16.8 | 0.363 |
| ^@^Contraceptive supplies (8 items) | 64.5 | 67.0 | 64.3 | 78.0* | **11.2*** | -2.2 | 20.5 | 0.049 |
| Electricity supply | 46.7 | 73.3* | 45.3 | 78.7* | **6.7** | -11.8 | 28.6 | 0.478 |
| Improved water source (piped, well, pump) | 37.3 | 81.3* | 33.3 | 93.3* | **16.0** | -6.3 | 38.5 | 0.154 |
| Functioning toilet (VIP or flush) | 73.3 | 93.3* | 65.3 | 98.7* | **13.3** | -0.3 | 26.5 | 0.055 |
| **Delivery care (as perceived by the mother)** |  |  |  |  |  |  |  |  |
| ^@^The facility was not dirty | 93.9 | 97.3* | 89.2 | 97.4* | **4.5** | -0.2 | 9.6 | 0.061 |
| ^@^The delivery room was clean | 95.4 | 97.6* | 93.8 | 98.4* | **2.7*** | 0.6 | 4.7 | 0.013 |
| ^@^The drugs the mother needed were available | 52.9 | 68.0* | 56.2 | 73.7* | **3.2** | -11.8 | 16.3 | 0.624 |
| The hours the facility is open were adequate | 94.2 | 93.9 | 88.8 | 96.2* | **8.3*** | 3.6 | 13.2 | 0.002 |
| **Any health service (as perceived by the mother)** | |  |  |  |  |  |  |  |
| ^@^Drugs available (last visit) | 30.6 | 35.0* | 34.2 | 54.4* | **16.2*** | 4.6 | 26.1 | 0.008 |
| Facility found open (any visit during past 2 years) | 84.0 | 85.9 | 82.1 | 86.8* | **2.7** | -3.8 | 10.0 | 0.419 |

^@^ Directly incentivized indicator.

*Significant at 0.05 level (baseline-endline comparison and DID estimation).

**Table D6a. Effects on process quality – communication (%).**

|  | **Comparison** | | **Intervention** | | **DID estimation (95% CI)** | | | |
| --- | --- | --- | --- | --- | --- | --- | --- | --- |
|  | **Base** | **End** | **Base** | **End** | **Estimate** | Lower | Upper | **p value** |
| **Antenatal care – communication** | |  |  |  |  |  |  |  |
| Discussed and advised on the place of delivery | 81.6 | 93.9* | 82.5 | 92.5* | **-1.6** | -7.0 | 4.0 | 0.571 |
| **Delivery care – communication** | |  |  |  |  |  |  |  |
| Staff introduced themselves | 19.7 | 31.5* | 16.6 | 37.5* | **9.5*** | 3.6 | 15.1 | 0.004 |
| Asked if the mother wanted someone to support her during delivery | 19.3 | 24.2* | 20.5 | 33.5* | **6.0*** | 1.9 | 10.5 | 0.003 |
| Explained what they were doing before conducting any procedure | 26.0 | 34.2* | 25.8 | 42.4* | **7.9** | -1.1 | 15.6 | 0.076 |
| Advised what to do to make the mother more comfortable during pain | 46.6 | 54.5* | 43.4 | 64.5* | **13.4*** | 5.5 | 21.9 | 0.001 |
| Did a good job at explaining the progress of the delivery | 77.9 | 89.3* | 78.3 | 91.3* | **1.6** | -2.4 | 6.0 | 0.395 |
| Discussed family planning | 29.3 | 54.0* | 33.3 | 55.2* | **-3.7** | -9.1 | 1.7 | 0.169 |
| Talked about danger signs | 17.8 | 36.2* | 22.0 | 49.7* | **8.0*** | 4.2 | 12.8 | <0.001 |
| Told the mother when to come back | 52.7 | 58.0* | 51.0 | 68.1* | **11.3** | -0.7 | 24.0 | 0.064 |
| Gave advice about breastfeeding | 35.7 | 53.6* | 35.9 | 60.2* | **3.5** | -4.6 | 11.8 | 0.378 |
| Discussed signs of newborn complications | 20.2 | 32.3* | 22.9 | 45.0* | **9.7*** | 3.6 | 15.8 | 0.003 |
| **Family planning – communication** |  |  |  |  |  |  |  |  |
| Explained how FP methods work | 76.9 | 85.7* | 70.0 | 85.1* | **4.4** | -4.4 | 13.5 | 0.289 |
| Explained the advantages and disadvantages of a particular method | 68.8 | 73.7* | 62.1 | 75.3* | **7.1** | -3.8 | 20.5 | 0.219 |
| When the method of choice was not available, health worker told where she could receive it | 83.8 | 97.2* | 87.6 | 96.5* | **-3.1** | -9.8 | 2.9 | 0.353 |
| Explained what to do in case of side effects | 49.6 | 63.3* | 45.7 | 70.5* | **10.5*** | 2.3 | 18.6 | 0.018 |

*Significant at 0.05 level (baseline-endline comparison and DID estimation).

**Table D6b. Effects on process quality – responsiveness (%, unless stated otherwise).**

|  | **Comparison** | | **Intervention** | | **DID estimation (95% CI)** | | | |
| --- | --- | --- | --- | --- | --- | --- | --- | --- |
|  | **Base** | **End** | **Base** | **End** | **Estimate** | Lower | Upper | **p value** |
| **Delivery care – responsiveness** |  |  |  |  |  |  |  |  |
| Time spent with the health provider during the delivery was not too low | 46.9 | 51.6* | 46.4 | 53.4* | **1.8** | -6.1 | 9.8 | 0.663 |
| Staff helped make the mother more comfortable during labour | 60.9 | 71.9* | 55.8 | 79.5* | **12.5*** | 4.6 | 21.1 | 0.004 |
| Staff came to assist the mother when she called for help | 75.4 | 87.8* | 74.8 | 86.6* | **0.3** | -5.4 | 6.3 | 0.898 |
| Privacy was sufficiently respected | 89.0 | 91.1 | 84.8 | 93.2* | **7.2*** | 0.8 | 13.7 | 0.029 |
| Was treated with respect and dignity | 49.0 | 89.0* | 49.1 | 81.5* | **-6.7** | -15.1 | 1.3 | 0.108 |
| Staff’s kindness (mean, rated from 0 to 100) | 76.3 | 84.4* | 71.4 | 84.4* | **5.0*** | 0.2 | 8.3 | 0.041 |
| **Any health service – responsiveness** |  |  |  |  |  |  |  |  |
| Staff took time to listen carefully | 87.4 | 90.3 | 83.5 | 93.5* | **7.7*** | 1.2 | 13.9 | 0.021 |
| No harsh words to the patients | 76.0 | 79.2* | 76.1 | 86.1* | **6.9*** | 0.7 | 14.1 | 0.03 |
| Treatment provided equally to rich and poor | 79.5 | 87.8* | 77.5 | 90.4* | **5.8** | -0.6 | 12.3 | 0.069 |
| Waiting time less than one hour (last visit) | 47.8 | 47.5 | 49.2 | 60.1* | **12.2*** | 4.7 | 19.7 | 0.008 |

*Significant at 0.05 level (baseline-endline comparison and DID estimation).

**Table D7. Effects on patient satisfaction (%).**

|  | **Comparison** | | **Intervention** | | **DID estimation (95% CI)** | | | |
| --- | --- | --- | --- | --- | --- | --- | --- | --- |
|  | **Base** | **End** | **Base** | **End** | **Estimate** | Lower | Upper | **p value** |
| **Delivery care** |  |  |  |  |  |  |  |  |
| The overall quality of the service was satisfactory^@^ | 88.0 | 92.2* | 84.8 | 94.7* | **5.4** | -0.3 | 10.9 | 0.057 |
| Mother would recommend the facility to friends | 91.5 | 95.2* | 90.6 | 96.3* | **2.3** | -1.8 | 6.3 | 0.253 |
| **Any health service** |  |  |  |  |  |  |  |  |
| Satisfied with the overall quality of the service^@^ | 66 | 71.3* | 67.9 | 83.4* | **10.9** | -2.1 | 22.5 | 0.096 |

^@^ Directly incentivized indicator.

*Significant at 0.05 level (baseline-endline comparison and DID estimation).

**Table D8. Effects on health worker job satisfaction and motivation (score on five-point Likert scale).**

|  | **Comparison** | | **Intervention** | | **DID estimation (95% CI)** | | | |
| --- | --- | --- | --- | --- | --- | --- | --- | --- |
|  | **Base** | **End** | **Base** | **End** | **Estimate** | Lower | Upper | **p value** |
| Satisfaction with working conditions (5 items)^a^ | 2.65  (0.81) | 3.24*  (0.88) | 2.80  (0.87) | 3.73*  (0.76) | **0.37** | -0.04 | 0.8 | 0.064 |
| Personal drive (6 items)^a^ | 3.32  (0.42) | 3.45*  (0.39) | 3.27  (0.39) | 3.44*  (0.42) | **0.05** | -0.02 | 0.17 | 0.173 |

**Table D11. Effects on service availability and co-payments (%, unless stated otherwise).**

|  | **Comparison** | | **Intervention** | | **DID estimation (95% CI)** | | | |
| --- | --- | --- | --- | --- | --- | --- | --- | --- |
|  | **Base** | **End** | **Base** | **End** | **Estimate** | Lower | Upper | **p value** |
| **Health service availability** |  |  |  |  |  |  |  |  |
| Days a week open for outpatient services (mean (sd)) | 5.3 (0.7) | 5.4 (0.8) | 5.4 (1.0) | 5.6 (0.9) | **0.1** | -0.3 | 0.5 | 0.534 |
| Facility offers 24 hours delivery services | 86.5 | 85.3 | 89.3 | 88.0 | **-0.2** | -15.4 | 15.1 | 0.981 |
| Facility conducts outreaches services | 69.3 | 94.7* | 78.7 | 98.7* | **-5.3** | -20.5 | 9.9 | 0.492 |
| **Co-payments: Delivery care** |  |  |  |  |  |  |  |  |
| Did not pay for delivery care services | 73.1 | 87.1* | 68.2 | 95.6* | **11.8*** | 2.7 | 21.1 | 0.015 |
| Did not purchase supplies to bring for the birth | 61.9 | 46.5* | 42.7 | 32.4* | **5.2** | -4.1 | 14.9 | 0.274 |

*Significant at 0.05 level (baseline-endline comparison and DID estimation).

**Table D12. Effects on Community Health Worker performance.**

|  | **Comparison** | | **Intervention** | | **DID estimation (95% CI)** | | | |
| --- | --- | --- | --- | --- | --- | --- | --- | --- |
|  | **Base** | **End** | **Base** | **End** | **Estimate** | Lower | Upper | **p value** |
| Number of women escorted last 3 months | 3.3  (7.7) | 7.3*  (7.5) | 2.5  (4.9) | 11.4*  (11.1) | **4.3** | -0.4 | 8.9 | 0.071 |
| Number of households visited last week | 13.3  (26.7) | 11.4  (15.5) | 13.0  (28.1) | 11.2  (11.0) | **0.8** | –3.9 | 3.4 | 0.829 |

*Significant at 0.05 level (baseline-endline comparison and DID estimation).
