# Supplementary material for: Does dosage matter? Effects of results-based financing layered on top of less comprehensive direct facility financing in Tanzania
Source: Health Policy Plan. 2026 May 13;41(6):988–1003. doi: 10.1093/heapol/czag058 (PMC13276258; doi:10.1093/heapol/czag058)
Supplement: Supplementary file 3 [file czag058_supplementary_data.zip › Supplementary Tables. Pre-trends.odt]

Supplementary Tables: Pre-trends. 

Table P1: Service utilization.

|  | DID estimate | SE | p-value | 95% CI |
| --- | --- | --- | --- | --- |
| Lower | Upper |
| Antenatal |  |  |  |  |  |
| ANC consultation before 12 weeks | 0.000197 | 0.000087 | 0.026* | 0.000024 | 0.000369 |
| At least 4 ANC visits | 0.000074 | 0.000098 | 0.453 | -0.000120 | 0.000267 |
| Delivery and postnatal |  |  |  |  |  |
| Institutional delivery | -0.000150 | 0.000094 | 0.113 | -0.000336 | 0.000036 |
| Postnatal check-up within 3-7 days | 0.000342 | 0.000205 | 0.098 | -0.000064 | 0.000747 |
| Childhood immunization and nutrition supplementation |  |  |  |  |  |
| Measles vaccination | 0.000006 | 0.000207 | 0.977 | -0.000403 | 0.000414 |
| Vitamin A supplementation | -0.000067 | 0.000130 | 0.608 | -0.000323 | 0.000190 |
| BCG vaccination | -0.000424 | 0.000086 | <0.001* | -0.000595 | -0.000254 |
| DPTHibHepB3 vaccination | -0.000070 | 0.000113 | 0.538 | -0.000293 | 0.000154 |
| Mebendazole | -0.000116 | 0.000095 | 0.223 | -0.000302 | 0.000071 |
| Family planning |  |  |  |  |  |
| Using any family planning method | 0.000019 | 0.000080 | 0.808 | -0.000138 | 0.000177 |

Table P2: Content of care.

|  | DID estimate | SE | p-value | 95% CI |
| --- | --- | --- | --- | --- |
| Lower | Upper |
| Antenatal care |  |  |  |  |  |
| Measured height | -0.000162 | 0.000085 | 0.058 | -0.000329 | 0.000006 |
| Took blood sample | -0.000108 | 0.000083 | 0.198 | -0.000272 | 0.000057 |
| Measured blood pressure | -0.000244 | 0.000097 | 0.013* | -0.000436 | -0.000052 |
| Listened to baby’s heart | -0.000027 | 0.000037 | 0.462 | -0.000100 | 0.000046 |
| Gave or prescribed iron or folic acid | 0.000062 | 0.000067 | 0.353 | -0.000070 | 0.000194 |
| Gave IPT, at least two doses | 0.000039 | 0.000124 | 0.752 | -0.000206 | 0.000285 |
| Gave tetanus vaccination | -0.000109 | 0.000079 | 0.173 | -0.000265 | 0.000048 |
| Gave Mebendazole | -0.000083 | 0.000103 | 0.421 | -0.000286 | 0.000120 |
| Measured weight | 0.000000 | 0.000053 | 0.998 | -0.000105 | 0.000105 |
| Analysed urine | -0.000078 | 0.000085 | 0.359 | -0.000246 | 0.000090 |
| Gave voucher for bednet | -0.000050 | 0.000090 | 0.581 | -0.000228 | 0.000128 |
| Delivery care - the mother |  |  |  |  |  |
| Checked blood pressure | -0.000075 | 0.000105 | 0.474 | -0.000283 | 0.000132 |
| Took blood test | -0.000027 | 0.000085 | 0.754 | -0.000194 | 0.000141 |
| Asked about abnormal bleeding | -0.000146 | 0.000093 | 0.120 | -0.000330 | 0.000038 |
| Examined abdomen | 0.000060 | 0.000119 | 0.617 | -0.000176 | 0.000296 |
| Examined breasts | 0.000003 | 0.000126 | 0.979 | -0.000245 | 0.000252 |
| Examined vagina | -0.000095 | 0.000117 | 0.421 | -0.000327 | 0.000137 |
| Delivery care - the infant |  |  |  |  |  |
| Weighed infant at birth | 0.000055 | 0.000053 | 0.302 | -0.000049 | 0.000159 |
| Breastfeeding within an hour | -0.000072 | 0.000093 | 0.443 | -0.000257 | 0.000113 |
| Vaccinated the infant | -0.000194 | 0.000147 | 0.191 | -0.000485 | 0.000097 |

Table P3: Structural quality.

|  | DID estimate | SE | p-value | 95% CI |
| --- | --- | --- | --- | --- |
| Lower | Upper |
| Delivery care (as perceived by the mother) |  |  |  |  |  |
| The facility was not dirty | -0.000051 | 0.000077 | 0.510 | -0.000203 | 0.000101 |
| The delivery room was clean | -0.000010 | 0.000063 | 0.877 | -0.000135 | 0.000115 |
| The drugs the mother needed were available | 0.000445 | 0.000175 | 0.012* | 0.000100 | 0.000790 |
| The hours the facility is open were adequate | 0.000080 | 0.000072 | 0.266 | -0.000062 | 0.000223 |
| Any health service (as perceived by the mother) |  |  |  |  |  |
| Drugs available (last visit) | 0.000014 | 0.000109 | 0.894 | -0.000201 | 0.000230 |
| Facility found open (any visit during past 2 years) | -0.000036 | 0.000081 | 0.652 | -0.000196 | 0.000123 |

Table P4a: Process quality – communication. 

|  | DID estimate | SE | p-value | 95% CI |
| --- | --- | --- | --- | --- |
| Lower | Upper |
| Antenatal care - communication |  |  |  |  |  |
| Discussed and advised on the place of delivery | -0.000036 | 0.000079 | 0.646 | -0.000193 | 0.000120 |
| Delivery care - communication |  |  |  |  |  |
| Staff introduced themselves | 0.000131 | 0.000112 | 0.244 | -0.000090 | 0.000352 |
| Asked if the mother wanted someone to support her during delivery | 0.000046 | 0.000112 | 0.679 | -0.000174 | 0.000267 |
| Explained what they were doing before conducting any procedure | 0.000004 | 0.000105 | 0.966 | -0.000203 | 0.000212 |
| Advised what to do to make the mother more comfortable during pain | -0.000134 | 0.000121 | 0.268 | -0.000372 | 0.000104 |
| Did a good job at explaining the progress of the delivery | -0.000166 | 0.000122 | 0.174 | -0.000406 | 0.000074 |
| Discussed family planning | -0.000102 | 0.000125 | 0.413 | -0.000349 | 0.000144 |
| Talked about danger signs | -0.000020 | 0.000099 | 0.843 | -0.000215 | 0.000176 |
| Told the mother when to come back | -0.000004 | 0.000126 | 0.977 | -0.000253 | 0.000245 |
| Gave advice about breastfeeding | -0.000114 | 0.000129 | 0.376 | -0.000368 | 0.000140 |
| Discussed signs of newborn complications | -0.000124 | 0.000104 | 0.238 | -0.000329 | 0.000082 |
| Family planning |  |  |  |  |  |
| Explained how FP methods work | -0.000062 | 0.000170 | 0.717 | -0.000398 | 0.000274 |
| Explained the advantages and disadvantages of a particular method | -0.000121 | 0.000158 | 0.447 | -0.000434 | 0.000192 |
| When the method of choice was not available, health worker told where she could receive it | 0.000054 | 0.000118 | 0.651 | -0.000180 | 0.000287 |
| Explained what to do in case of side effects | 0.000132 | 0.000149 | 0.378 | -0.000163 | 0.000427 |

Table P4b: Process quality – responsiveness. 

|  | DID estimate | SE | p-value | 95% CI |
| --- | --- | --- | --- | --- |
| Lower | Upper |
| Delivery care - staff responsiveness |  |  |  |  |  |
| Time spent with the health provider during the delivery was not too low | -0.000171 | 0.000137 | 0.214 | -0.000442 | 0.000100 |
| Staff helped make the mother more comfortable during labour | -0.000155 | 0.000131 | 0.237 | -0.000414 | 0.000103 |
| Staff came to assist the mother when she called for help | -0.000154 | 0.000116 | 0.187 | -0.000383 | 0.000075 |
| Privacy was sufficiently respected | 0.000030 | 0.000089 | 0.734 | -0.000146 | 0.000207 |
| Was treated with respect and dignity | 0.000058 | 0.000128 | 0.649 | -0.000194 | 0.000311 |
| Staff’s kindness | -0.000670 | 0.000562 | 0.235 | -0.001780 | 0.000440 |
| Any health service - staff responsiveness |  |  |  |  |  |
| Staff took time to listen carefully | -0.000092 | 0.000070 | 0.192 | -0.000230 | 0.000046 |
| No harsh words to the patients | 0.000019 | 0.000083 | 0.820 | -0.000146 | 0.000184 |
| Treatment provided equally to rich and poor | -0.000013 | 0.000081 | 0.872 | -0.000172 | 0.000146 |
| Waiting time less than one hour (last visit) | -0.000003 | 0.000100 | 0.976 | -0.000200 | 0.000194 |

Table P5: Patient satisfaction.

|  | DID estimate | SE | p-value | 95% CI |
| --- | --- | --- | --- | --- |
| Lower | Upper |
| Delivery care |  |  |  |  |  |
| The overall quality of the service received was satisfactory | -0.000092 | 0.000090 | 0.306 | -0.000270 | 0.000085 |
| Mother would recommend the facility to friends | 0.000013 | 0.000081 | 0.875 | -0.000148 | 0.000174 |
| Any health service |  |  |  |  |  |
| Satisfied with overall quality of the service | -0.000113 | 0.000093 | 0.226 | -0.000298 | 0.000071 |
